# Mical modulates Tau toxicity via cysteine oxidation in vivo

**DOI:** 10.1186/s40478-022-01348-1

**Published:** 2022-04-04

**Authors:** Engie Prifti, Eleni N. Tsakiri, Ergina Vourkou, George Stamatakis, Martina Samiotaki, Efthimios M. C. Skoulakis, Katerina Papanikolopoulou

**Affiliations:** 1 Institute for Fundamental Biomedical Research, Biomedical Sciences Research Centre “Alexander Fleming”, 34 Fleming Street, 16672 Vari, Greece; 2 Institute for Bio-Innovation, Biomedical Sciences Research Centre “Alexander Fleming”, 34 Fleming Street, 16672 Vari, Greece; 3 Laboratory of Genetics, Department of Biotechnology, Agricultural University of Athens, Iera Odos 75, 11855 Athens, Greece

**Keywords:** Tau protein, Mical, Toxicity, Aggregation, Cysteines, Post-translational modification, Oxidation

## Abstract

Tau accumulation is clearly linked to pathogenesis in Alzheimer’s disease and other Tauopathies. However, processes leading to Tau fibrillization and reasons for its pathogenicity remain largely elusive. Mical emerged as a novel interacting protein of human Tau expressed in Drosophila brains. Mical is characterized by the presence of a flavoprotein monooxygenase domain that generates redox potential with which it can oxidize target proteins. In the well-established Drosophila Tauopathy model, we use genetic interactions to show that Mical alters Tau interactions with microtubules and the Actin cytoskeleton and greatly affects Tau aggregation propensity and Tau-associated toxicity and dysfunction. Exploration of the mechanism was pursued using a Mical inhibitor, a mutation in Mical that selectively disrupts its monooxygenase domain, Tau transgenes mutated at cysteine residues targeted by Mical and mass spectrometry analysis to quantify cysteine oxidation. The collective evidence strongly indicates that Mical’s redox activity mediates the effects on Tau via oxidation of Cys322. Importantly, we also validate results from the fly model in human Tauopathy samples by showing that MICAL1 is up-regulated in patient brains and co-localizes with Tau in Pick bodies. Our work provides mechanistic insights into the role of the Tau cysteine residues as redox-switches regulating the process of Tau self-assembly into inclusions in vivo, its function as a cytoskeletal protein and its effect on neuronal toxicity and dysfunction.

**Supplementary Information:**

The online version contains supplementary material available at 10.1186/s40478-022-01348-1.

## Introduction

In Alzheimer’s disease (AD) and related Tauopathies, aggregation of abnormally phosphorylated Tau protein is considered central to disease pathogenesis. Tauopathies encompass a range of neurodegenerative disorders including Pick’s disease (PiD) and Frontotemporal Dementia (FTD), presenting varying clinical symptoms depending on the type of lesion, cell type and affected brain region [1]. Tau is primarily distributed in axons of the central nervous system (CNS) where it plays major roles in the regulation of microtubule dynamics and axonal transport [2]. Several studies have identified multiple physiological functions of Tau at the postsynaptic compartment and in the nucleus, as well as interactions with mitochondria, the plasma membrane and the Actin cytoskeleton [3]. In the adult human brain alternative splicing of a single-copy gene generates six Tau isoforms that differ by the absence or presence of one or two inserts in the amino-terminal part (0 N, 1 N or 2 N), in combination with either three or four imperfect repeats (3R or 4R) that possess the microtubule binding activity of the protein, in the carboxy-terminal part. The affinity of Tau for microtubules depends on the number of repeats and the degree of its phosphorylation. Isoforms with four repeats (4R) bind to microtubules with a greater affinity than 3R species and extensive phosphorylation reduces binding efficiency [4, 5].

Post-translationally modified Tau is the primary component of intracellular aggregates, a pathological hallmark of AD and other Tauopathies [1, 6]. Although phosphorylation at both physiological and non-physiological sites is the most extensively studied Tau post-translational modification (PTM), the protein undergoes additional such modifications including acetylation, ubiquitination, glycosylation and oxidation [6, 7]. Tau presents high conformational flexibility primarily arising from the excess of polar amino acids, the relatively small fraction of bulky hydrophobic amino acid side-chains and its high proline content [6, 8]. This unusual amino acid composition renders it highly soluble and it is therefore unconventional that it assembles into filaments. Under pathological conditions, PTMs are thought to alter the conformation of Tau by acting locally or distantly from the site of modification, which may modulate the propensity for formation of different fibrillar structures [9]. In addition, two hexapeptide motifs within the microtubule-binding repeat region have been shown to possess high β-sheet-forming propensity, promoting aggregation and triggering pathogenicity [10, 11].

Protein conformation is also sensitive to reduction–oxidation (redox) changes with cysteine residues being the main target of such oxidative modifications. Because oxidation is reversible at physiological conditions, cysteines can act as a powerful molecular switch regulating the function, binding interactions and conformation of the protein. Human Tau possesses two cysteine residues, within the microtubule binding domain: Cys291 and Cys322. Cys322 is present in all six human Tau isoforms, whereas Cys291 is present only in 4R isoforms. These appear capable of forming disulfide linked dimers that can serve as templates to accelerate the conformational conversion of Tau into insoluble fibrillary aggregates in vitro [12–15] and compounds that target these residues and inhibit oxidation prevent aggregation [16–18]. However, the consequences of oxidation on Tau function and aggregation propensity in vivo remain elusive.

To identify proteins engaged in pathways leading to Tau pathology, we employed a proteomic approach to define proteins that interact with Tau in the Drosophila CNS yielding Mical among others. MICALs (Molecules Interacting with CasL) comprise a family of phylogenetically conserved multi-domain flavoprotein monooxygenases. MICALs may interact with multiple different proteins and affect them functionally through redox modifications [19]. Vertebrate MICALs have been implicated in axon guidance, exocytosis, apoptosis and CNS regeneration [20]. In Drosophila, Mical through its intrinsic redox activity oxidizes Actin resulting in disassembly of Actin filaments [21, 22] and is required for axon pathfinding, synaptic bouton redistribution and denditric pruning [23, 24]. MICALs control the function of their target proteins via redox modification of cysteine and methionine side chains leading to formation of disulfides or methionine oxidation respectively [25].

As we are interested in understanding the nature of the interaction between Tau and Mical and given the importance of cysteine residues for specific physiological and patho-physiological functions of Tau [7], we hypothesized that they are the most likely candidate amino acids to be post-translationally modified by the enzyme. We describe the genetic interactions between the two proteins with respect to Tau normal function as a cytoskeletal protein, Tau-associated toxicity and dysfunction. Finally, using mass spectrometry analysis we identify Cys322 oxidation and we provide insights into how Tau can adopt a highly aggregated conformation in vivo.

## Materials and methods

### Drosophila culture and strains

Flies were cultured in standard sugar-wheat flour food supplemented with soy flour and CaCl_2_ [26]. Panneuronal transgene expression was achieved using the *elav*^*C155*^*-GAL4* or the *elav*^*C155*^*-GAL4;Ras2-GAL4* double driver as already described [7, 27]. Fly crosses and experiments were performed at 25 °C unless noted otherwise. The *elav*^*C155*^*-GAL4;tub-Gal80ts* strain was constructed using standard methods [28] and was used in order to prevent expression during development. The fly line carrying UAS-htau^0N4R^ was a gift of Dr. M. Feany (Harvard Medical School, [29]) and UAS-hTau^0N3R^ of Dr. Stefan Thor (Linkoping University, [30]). The generation of UAS-htau^FLAG−2N4R^, UAS-htau^FLAG−2N4RC291A^ and UAS-htau^FLAG−2N4RC322A^ transgenes has been described previously in [7, 31]. Fly lines carrying UAS transgenes of full-length GFPMical and Mical G → W mutation (MicalΔredox) were kindly provided by Dr. Jonathan Terman (University of Texas Southwestern Medical Center) [22, 32]. Two different UAS-Mical RNAi-mediating transgenes were used. The first was obtained from the National Institute of Genetics (NIG) in Japan (18668R-2) and the second (ID25372, described in [23]) was a kind gift from Dr. Hermann Aberle (Heinrich-Heine-University). All the flies were backcrossed into the resident Cantonized *w*^*1188*^ control background for five generations. Double strains with Tau and Mical transgenes together with a CyO balancer [33] were generated by standard genetic methods.

### LC–MS/MS analysis

The method is described in detail in [7]. Three biological and three to four technical replicas from each genotype were used for this experiment. Briefly, flies expressing paneuronally htau^FLAG−2N4R^ alone and upon co-expression with Mical or Mical RNAi (18668R-2) under *elav*^*C155*^*-GAL4;Ras2-GAL4* were decapitated *en masse* by sieving in liquid nitrogen. Upon homogenization of the harvested heads in lysis buffer consisting of 50 mM Tris HCl, pH 7.4, 150 mM NaCl, 1 mM EDTA, 1% Triton X-100 supplemented with protease and phosphatase inhibitors, supernatants were incubated overnight at 4 °C with anti-FLAG coated agarose beads (Sigma) using a roller shaker. Elution was performed under acidic conditions using 0.1 M glycine HCl, pH 3.5 and eluent samples were subjected to the Sp3 protein purification and digestion protocol [34]. Peptide products were analyzed by nano-LC–MS/MS using a Q Exactive Orbitrap HF-X mass spectrometer (Thermo Fisher Scientific, Waltham, MA, USA).

The raw output files were analyzed using MaxQuant software (1.6.17.0) [35] against the complete Uniprot proteome of *Drosophila melanogaster* (Downloaded 17 February 2020/22,045 entries) and a common contaminants database by the Andromeda search engine. Protein abundance was calculated on the basis of the normalized spectral protein intensity as label free quantitation (LFQ intensity).

### Targeted proteomics

Three biological and two technical replicas from each genotype were used for this experiment. Flies expressing panneuronally htau^FLAG−2N4R^ alone or upon co-expression with Mical under *elav*^*C155*^*-GAL4;Ras2-GAL4* were decapitated *en masse* by sieving in liquid nitrogen. Upon homogenization of the harvested heads in lysis buffer consisting of 50 mM Hepes, pH 7.5, 150 mM NaCl, 0.25% Triton X-100, 20 mM N-ethyl maleimide (NEM) supplemented with protease and phosphatase inhibitors, supernatants were incubated for two hours at room temperature with anti-FLAG coated agarose beads (Sigma) using a roller shaker. Elution was performed under acidic conditions using 0.1 M glycine HCl, pH 3.5 and eluent samples were subjected to the Sp3 protein purification and digestion protocol [34]. During this protocol oxidized cysteines (unlabeled by NEM) were reduced with 100 mM Dithiothreitol (DTT) at 56 °C and alkylated in the dark with 200 mM iodoacetamide. Generated tryptic peptides were separated using nanoLC and analyzed by Data Independent Acquisition (DIA) using a 60 min gradient and 39 deconvoluted windows of 8 m/z ranging from 370 to 1000 m/z on a Q Exactive HF-X Orbitrap instrument. The raw files were imported into Skyline-daily 21.2.1.403 [36] using the peptide search pipeline. A library was build using DIA-UMPIRE and MSFragger 3.4 [37] for peptides from 350 to 1100 m/z and 2–4 charges against the *Drosophila melanogaster* proteome and Human Tau isoform 2N4R. The NEM (+ 125.1253 Da) and carbamidomethyl (+ 57.0513 Da) were used as variable modifications. The results are expressed as the ratio of the area of the cysteine containing peptide (^322^**C**GSLGNIHHKPGGGQVEVK) modified by NEM *versus* the modified by carbamidomethyl counterpart.

### Western blotting, pull-downs and antibodies

For western blotting, adult fly heads at 1–3 days post-eclosion were homogenized in 1 × Laemmli buffer (50 mM Tris pH 6.8, 100 mM DTT, 5% 2-mercaptoethanol, 2% SDS, 10% glycerol and 0.01% bromophenol blue), the extracts heated for 3 min at 95 °C, centrifuged at 11,000*g* for 5 min and separated in 10% SDS-acrylamide gels.

For the pull-downs, experiments were performed as described in LC–MS/MS but instead of eluting the samples with 0.1 M glycine HCl, pH 3.5 beads were mixed with 1 × Laemmli buffer without 2-mercaptoethanol, in order to minimize the denaturation and elution of the FLAG antibody.

Proteins were transferred to PVDF membranes and probed with mouse monoclonal anti-Tau (5A6, Developmental Studies Hybridoma Bank), AT100, AT270 and AT8 from Thermo Fischer Scientific and the polyclonal antibodies anti-pS262 (ProSci) and anti-pS396 (Cell Signaling). All Tau antibodies were used at 1:1000 whereas the appropriate anti-mouse or anti-rabbit HRP-conjugated secondary antibody was applied at 1:5000 dilution. The Mical antibody was a kind gift from Dr. Jonathan Terman [32] and was used at 1:1000 dilution. The rabbit polyclonal anti-dTau [38] was from Dr. Nick Lowe (Cambridge University, UK) and was used at 1:2000. Chicken polyclonal anti-14-3-3 epsilon was used at 1:2000 [39]. To normalize for sample loading, the membranes were concurrently probed with an anti-Syntaxin primary antibody (8C3, Developmental Studies Hybridoma Bank) at 1:3000 dilution.

Human tissues obtained from Netherlands Brain Bank (NBB), Netherlands Institute for Neuroscience, Amsterdam, were used as per MTA 457.13. All materials have been collected from donors under written informed consent for a brain autopsy and the use of the material and clinical information for research purposes obtained by the NBB [40]. Human brain tissue samples were homogenized in Laemmli buffer (1:10) containing 5% of SDS, aliquoted and frozen at − 80 °C until used [40, 41]. After separation in 10% SDS-acrylamide gels, membranes have been probed with anti-MICAL1 (1:250, Proteintech) and anti-Actin (1:1000, Sigma). Proteins were visualized with chemiluminescence (Immobilon Crescendo, Millipore) and signals were quantified by densitometry with the Image Lab 5.2 program (BioRad).

### Microtubule-binding assay

Microtubule-binding experiments described in detail in [7, 42] determine Tau binding to Taxol-stabilized microtubules isolated from fly head extracts or to exogenously added bovine microtubules (Cytoskeleton, Denver, CO, USA). After ultracentrifugation at 100,000*g* supernatant and pellet fractions were collected and analyzed by immunoblotting with anti-Tau 5A6 and E7 beta-Tubulin at 1:1000 dilution from Developmental Studies Hybridoma Bank to estimate the amount of Tau bound to microtubules.

### F-Actin precipitation assay

Total F-Actin has been isolated as in [7, 42]. Briefly, biotinylated phalloidin (Invitrogen, Molecular Probes) was added to 8 fly brains from each genotype, homogenized in 25 μl of 100 mM Na_2_HPO4–NaH_2_PO4 at pH 7.2, 2 mM ATP, 2 mM MgCl_2_ supplemented with phosphatase (Sigma) and protease (Thermo Scientific) inhibitor cocktails. After incubation with streptavidin-coupled Dynabeads (Invitrogen), the precipitated material and supernatant were probed with 5A6 (1:1000) and anti-Actin (1:1000, Sigma).

### Tau solubility assay

For the extraction of aggregates with formic acid as described in [7, 40], flies were raised at 25 °C and then aged for 10 days at 30 °C. Upon homogenization of fly heads in RIPA buffer (50 mM Tris–HCl pH 8.0, 150 mM NaCl, 20 mM EDTA, 1% Nonidet-P40 supplemented with protease and phosphatase inhibitors) and centrifugation at 11,300*g* for 20 min at 4 °C, pellets were treated with 70% formic acid (FA). RIPA and FA fractions were separated by SDS-PAGE and analyzed by immunoblotting.

For the extraction of aggregates with SDS as described in [7, 43], fly heads were homogenized in 50 mM Tris–HCl pH 7.4, 175 mM NaCl, 1 M sucrose, 5 mM EDTA supplemented with protease and phosphatase inhibitors. The samples were then spun for 2 min at 1000 g and the supernatant was centrifuged at 186,000*g* for 2 h at 4 °C. The supernatants were regarded as the soluble fraction and the resulting pellets were re-suspended in SDS buffer (50 mM Tris–HCl pH 7.4, 175 mM NaCl, 5% SDS) and centrifuged for 2 h at 200,000*g* (25 °C). The supernatants were collected as the SDS-soluble fraction. Soluble and insoluble fractions from both assays were separated by SDS-PAGE and analyzed by immunoblotting as described previously [7].

### Lifespan determination

Animals expressing Tau and Mical transgenes under *elav*^*C155*^*-GAL4; tub-Gal80ts* were raised at 18 °C together with control single copy driver flies. Groups of 20 young flies (10 males and 10 females, 1–3 days old) were collected and maintained at 29 °C until they expired. Flies were transferred to fresh vials every 3 days. Experiments have independently been performed with groups of 20 males. At least 300 flies were assessed per genotype.

### Paraquat sensitivity

Animals expressing panneuronally Tau and Mical transgenes were raised at 25 °C together with control single copy driver flies. Paraquat feeding has been performed as described in [7, 27]. Groups of 20 flies (10 males and 10 females, 1–3 days old) were treated with 30 mM of methyl viologen (Acros Organics) supplemented in standard fly food. At least 300 flies were assessed per genotype.

### Viability assays

To determine the effect of Tau and Mical expression on viability, 5 transgenic Tau females were crossed with 3 *elav*^*C155*^*-GAL4* males (elav is on the X chromosome), or *elav*^*C155*^*- GAL4*;UAS-Mic/CyO and *elav*^*C155*^*-GAL4*;UAS-MicRNAi/CyO. After 24 h they were transferred to new vials and allowed to lay eggs for three days and then discarded. The number of non-CyO females *versus* non-CyO males was determined when adults emerged. Each assessment was performed at least in pentaplicate with five females each. Concomitantly 5 *w*^*1118*^ females were crossed with 3 *elav*^*C155*^*-GAL4* males and the female *versus* male ratio of their progeny was considered as control. *w*^*1118*^ females were also crossed with the above mentioned Mical Transgene *elav*^*C155*^*-GAL4* males to determine the effect of the two Mical transgenes on fly survival. For the drug experiments, crosses were performed using standard fly food supplement with 10 μM Methylene Blue (Sigma) or the indicated concentrations of green tea polyphenol (−)-epigallocatechin gallate EGCG (Fluorochem).

Viability assays were also performed by crossing 5 *elav*^*C155*^*-GAL4;Ras2-GAL4* females with 3 males UAS-Mic/CyO;UAS-C322A. The number of non-CyO flies expressing both Tau and Mical *versus* CyO flies expressing only C322A Tau was determined when adults emerged. Concomitantly 5 *elav*^*C155*^*-GAL4;Ras2-GAL4* females were crossed with UAS-Mic/CyO males and the non-CyO (expressing Mical) *versus* CyO (expressing no Mical) ratio of their progeny was considered as control.

### Behavioral analyses

Animals expressing UAS-htau^0N4R^ and UAS-Mical RNAi single and double transgenes under the control of the panneuronal *elav*^*C155*^*-GAL4;tub-Gal80ts* driver were raised at 18 °C together with control single copy driver flies. Conditional transgene expression under this driver was induced specifically in adult flies by incubation at 29 °C for 12 days post-emergence. Flies expressing panneuronally wild-type Tau (UAS-hTau^0N3R^, UAS-htau^FLAG−2N4R^), cysteine mutants and UAS-GFPMical transgenes were raised at 25 °C. All the progeny were separated in groups of 50–70 mixed sex animals and trained in classical olfactory aversive conditioning [44] as previously described [7, 42]. The aversive odors used for conditioning were benzaldehyde (6% *v*/v) and 3-octanol (50% *v*/v) diluted in isopropylmyristate (Fluka). Training and testing were carried out at 25 °C and 75% relative humidity under dim red light. For Long Term Memory (LTM) analyses, flies underwent 5 training cycles spaced at 15 min rest intervals, and tested 24 h later. Each training cycle consists of 1 min to odor A paired with twelve 90 V electric shocks at 5 s inter-stimulus interval, followed by 30 s of air and 1 min odor B without reinforcement. All experiments were carried out in a balanced manner, where all genotypes involved in an experiment were tested per day.

### Confocal microscopy

For immunofluorescence on human hippocampal brain tissue obtained from NBB, 8 μm formalin-fixed paraffin-embedded sections were de-paraffinized in xylene, rehydrated and boiled for 10 min in 10 mM Sodium Citrate buffer, 0.05% Tween-20, pH 6.0. After washing in Phosphate Buffered Saline (PBS, 137 mM NaCl, 2.7 mM KCl, 10 mM Na_2_HPO_4_, 1.8 mM KH_2_PO_4_, pH 7.4) they were incubated for 5 min in potassium-permanganate (0.25% in PBS, Fluka), washed and treated with a solution of potassium metabisulfite (2%, Sigma) and oxalic acid (1%, Acros Organics) until the brown color was removed from the tissue. Subsequently, all sections were washed with PBS and incubated for 3 h at room temperature in 2% BSA and 0.25% Tween-20 in PBS to block nonspecific binding. All primary antibodies, diluted in PBS containing 0.25% Tween-20 and 1% BSA, were applied to sections and then incubated overnight at 4 °C (AT8 1:500 and anti-Mical1 1:100 dilution). After rinsing in PBS, sections were incubated for 2 h at room temperature with a goat anti-rabbit antibody coupled to Alexa Fluor 488 and a goat anti-mouse coupled to Alexa Fluor 555 (1:700, Molecular Probes). Counterstaining of the nuclei was done with DAPI. Images were acquired at 40 × or 60 × with a zoom factor of 1 by laser-scanning confocal microscopy (TCS SP8, Leica). Pearson’s correlation coefficient (PCC) was measured using Just Another Colocalization Plugin JaCoP for ImageJ [45] from 7 independent images.

### Statistical analysis

Quantification of all Western blots was performed by densitometry and the ratio of a given protein relative to that of Syntaxin, Tubulin or Actin was calculated. The ratio of the control genotype was set to 1 and all experimental ratios were reported as relative to that. Results were plotted as means ± Standard Error of the Mean (SEM) from at least three independent experiments. The data were analyzed by standard parametric statistics using Dunnett’s tests relative to the designated control.

For the proteomic experiments, the statistical analysis of the LFQ intensities was performed with Perseus (version 1.6.10.43) using a two samples t-test with a false discovery rate (FDR) value of 0.05 [46]. Means and SEMs of peptide ratios NEM/carbamidomethyl were compared to that of designated control using Dunnett’s.

Means and SEMs of viability and survival upon oxidative stress toxicity were compared to that of designated control using Dunnett’s. Survival curves were compared using log-rank tests (JMP 7.1 statistical software package, SAS Institute Inc). Finally, memory performance indices calculated for each genotype were examined for differences using ANOVA, followed by planned multiple comparisons using the Least Squares Means (LSM) approach. Data were analyzed parametrically with the JMP statistical package (SAS Institute Inc., Cary, NC) as described before [42, 43].

## Results

### Mical alters the interaction properties of Tau

**Fig. 1:**
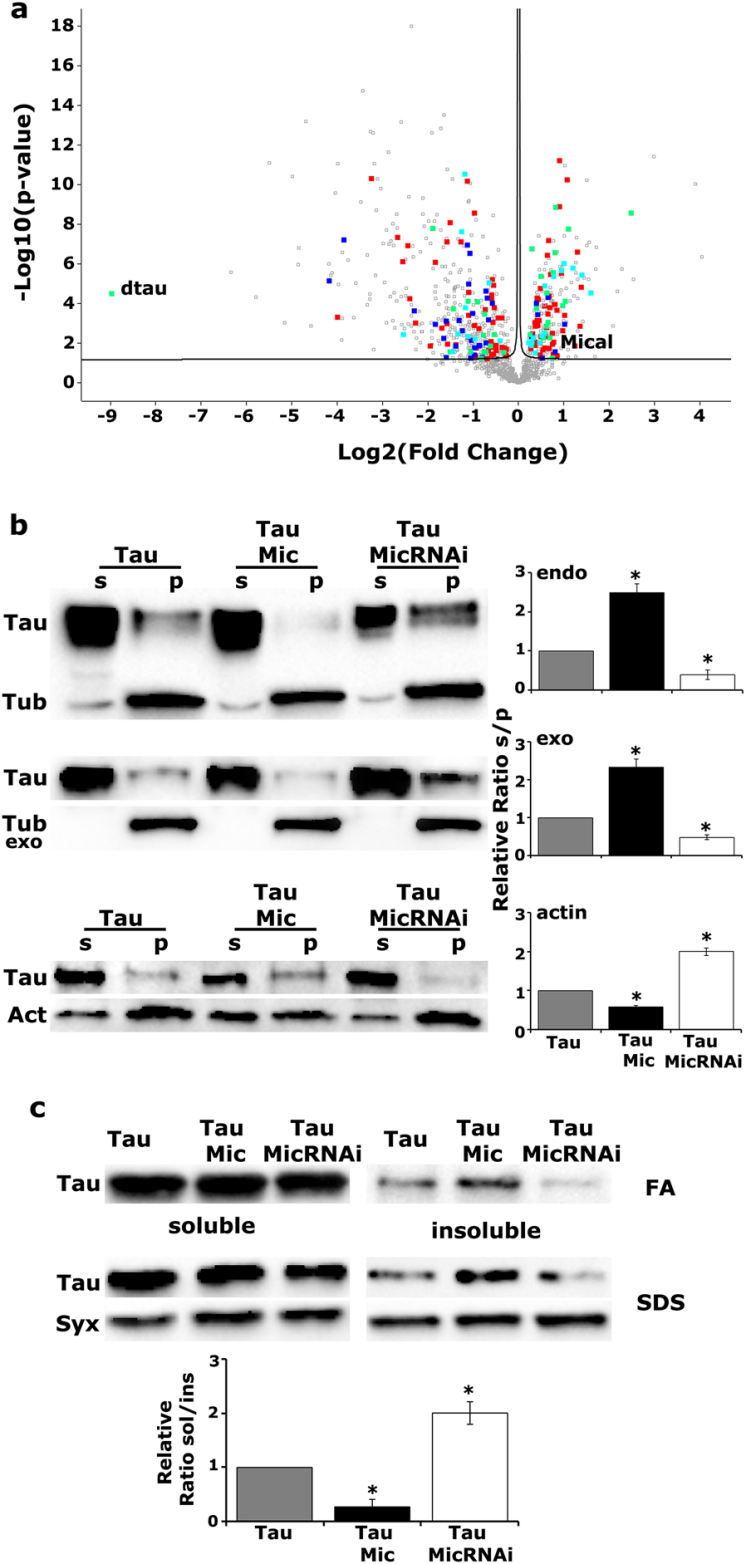
Mical precipitates global changes in the interactome of Tau. **a** Volcano plot of the log2 fold change *versus* the − log10 (p-value) representing the differential interactions of Tau upon co-overexpression with Mical. Proteins involved in microtubule cytoskeleton organization are highlighted in green, Actin cytoskeleton organization in blue, oxidation–reduction processes in red and synaptic transmission in cyan. **b** Endogenous microtubules (upper panel), preformed bovine microtubules (middle panel) or phalloidin-bound F-Actin (lower panel) have been isolated from lysates expressing under the *elav*^*C155*^*-GAL4* driver the hTau^0N4R^ transgene alone or upon Mical up and downregulation. p: pellet and s: supernatant fractions were analyzed by western blotting. Stars indicate significantly altered levels of precipitated Tau upon modulation of Mical levels compared to Tau expressed alone. **c** Proteins from adult heads following panneuronal expression of the indicated transgenes were sequentially extracted with RIPA buffer and 70% FA and probed for Tau (5A6). Alternatively, aqueous soluble and SDS soluble fractions were probed for Tau using Syntaxin (Syx) as loading control. The bars represent the mean ± SEM relative sol/ins ratios of Tau upon Mical up or down-regulation, over that of Tau alone. Stars indicate significantly altered solubility ratios

Co-immunoprecipitation coupled with mass spectrometry is an extremely powerful analytical technique and has been pivotal in protein–protein interaction studies [47]. Mical was discovered in an exploratory proteomic experiment of human Tau expressed in the Drosophila CNS and the interaction was independently confirmed by Western blot analysis (Additional file 2: Fig. S1a). To further validate the Tau-Mical interaction in vivo, we investigated whether overexpression of Mical changes the interactome of human Tau and applied a quantitative, label-free proteomic approach that allows an unbiased comparison of multiple samples [48, 49]. Using these high accuracy proteomics tools, we quantified the interactome of human Tau expressed pan-neuronally alone or upon co-overexpression with Mical. Proteins associated with Flag-tagged Tau were isolated using agarose beads coated with anti-Flag antibody followed by LC–MS analysis. A total of 1200 proteins were identified, out of which 690 were differentially abundant (*FDR* ≤ 0.05) between the two conditions. MICALs are considered to be unique among proteins involved in cytoskeleton dynamics in that they establish a direct link between redox signaling and cytoskeleton rearrangements [20, 50, 51]. Indeed, among the differentially abundant interactors, overexpression of Mical precipitated major changes in proteins implicated in microtubule cytoskeleton organization (Fig. 1a highlighted in green and Additional file 1: Table S1), Actin cytoskeleton organization (Fig. 1a in blue and Additional file 1: Table S2), oxidation–reduction processes (Fig. 1a in red and Additional file 1: Table S3) and synaptic transmission (Fig. 1a in cyan and Additional file 1: Table S4).

As expected, Mical was found on the right side of the volcano plot where proteins with increased abundances and/or increased affinity for Tau upon Mical excess are located. It is interesting to note that 14–3-3 epsilon, an established Tau interactor known to regulate its toxicity in vivo ([39], Additional file 1: Table S1), was also enriched on the right side of the plot. As an independent confirmation, elevated Mical greatly increased the levels of 14–3-3 epsilon (Additional file 2: Fig. S1b, p = 4.43e−05, n = 4) apparently leading to its enrichment as a Tau interactor.

Interestingly, among the differential interactors dTau presented the largest fold change (Fig. 1a and Additional file 1: Table S1). As shown in Additional file 2: Fig. S1c, d, dTau indeed co-precipitated with hTau, but in contrast to 14–3–3 epsilon, its expression was not altered upon Mical elevation (Additional file 2: Fig. S1c, p = 0.6610, n = 3). We thus wondered whether the differential interaction between hTau and dTau, as well as other cytoskeletal proteins, reflected differential cytoskeletal properties of hTau upon modulation of Mical levels.

To test this hypothesis, microtubule-binding affinity of hTau^0N4R^ was assessed upon Mical overexpression or its RNAi-mediated down-regulation. Endogenous microtubules were sedimented by ultracentrifugation from fly head lysates in the presence of the stabilizing agent Taxol (Fig. 1b, upper panel). In parallel, the lysates were incubated with preformed Taxol-stabilized bovine microtubules (Fig. 1b, middle panel). The pellet and supernatant fractions were subsequently probed for Tau and Tubulin. Quantification of three independent experiments representing the relative level of Tau in the supernatant and pellet fractions revealed that Mical up-regulation decreased the affinity of Tau for microtubules whereas its down-regulation enhanced their interaction (Fig. 1b, upper panel TauMic p = 0.0003, TauMicRNAi p = 0.0268, n = 3 and middle panel TauMic p = 0.0004, TauMicRNAi p = 0.0392, n = 3). Strikingly, when F-Actin was isolated from fresh brain extracts using biotinylated-phalloidin we observed the opposite effect. Quantitative analysis from three independent experiments representing the relative ratio of Tau in the supernatant and pellet fractions revealed that a significant fraction of Tau co-precipitated with F-Actin upon Mical up-regulation whereas upon Mical reduction the binding of Tau to F-Actin was greatly reduced (Fig. 1b, lower panel TauMic p = 0.0067, TauMicRNAi p = 2.1746e−05, n = 3).

Because down-regulation of Mical enhanced the interaction of Tau with microtubules and attenuated its interaction with F-Actin whereas its up-regulation had the opposite effect, we hypothesized that attenuation of Mical levels could also impact the interaction of Tau with other cytoskeletal proteins. By adopting a similar quantitative, label-free proteomic approach as the one described above, we identified the differentially abundant (*FDR* ≤ *0.05*) cytoskeletal proteins in extracts from flies expressing Tau alone or upon Mical RNAi-mediated attenuation, and compared them with those presented in Additional file 1: Tables S1 and S2. Interestingly, we identified many interactors with opposite abundances between the two conditions (Additional file 1: Table S5). For example, in contrast to Mical up-regulation, the interaction between dTau and hTau was highly enriched upon Mical attenuation, probably reflecting higher affinity of hTau for microtubules. It should be noted that Mical down-regulation had no effect on the expression levels of dTau (Additional file 2: Fig. S1c, p = 0.9862, n = 3), on the levels of 14–3–3 epsilon Additional file 2: Fig. S1b, p = 0.9549, n = 4) as well as on the interaction between Tau and 14–3–3 3 epsilon (Additional file 1: Table S5, p > 0.05).

Since the cytoskeletal interactions of Tau depend critically on the phosphorylation status of the protein [6], we examined whether alteration of Mical levels affects Tau phosphorylation pattern. Therefore, Tau phosphorylation in adult fly brains was assessed under endogenous, increased and attenuated Mical levels with a panel of antibodies targeting specific phosphorylated sites (Additional file 2: Fig. S1e). These phospho-antibodies recognize sites that are reported to be highly enriched in AD brains and include pS202/pT205 (AT8), pT212/pS214 (AT100), pS262, pT181 (AT270) and pS396. Interestingly, Mical dosage does not appear to affect neither Tau levels nor occupation of any of the phospho-sites tested (Additional file 2: Fig. S1e, **Total**: TauMic p = 0.3369, TauMicRNAi p = 0.5938, **pS396**: TauMic p = 0.9211, TauMicRNAi p = 0.8256, **AT8**: TauMic p = 0.6037, TauMicRNAi p = 0.4697, **AT100**: TauMic p = 0.3711, TauMicRNAi p = 0.9599, **AT270**: TauMic p = 0.8107, TauMicRNAi p = 0.7593, **pS262**: TauMic p = 0.8521, TauMicRNAi p = 0.7884, n = 4).

In addition, Tau solubility was assessed as another biochemical property of the protein that could in principle be affected upon Mical excess or attenuation. Head lysates from animals were fractionated into soluble and insoluble materials and were probed for Tau. Interestingly, Mical excess greatly increased the accumulation of Tau in the insoluble fraction, whereas the aggregation propensity of the protein was reduced when Mical levels were low as indicated by the ratio of soluble/insoluble fraction (Fig. 1c, TauMic p = 0.0090, TauMicRNAi p = 0.0020, n = 3). Collectively these results strongly suggest that upon increase of Mical levels, Tau exhibits biochemical properties that lead to its aggregation, and could then also underlie its toxicity.

### Excess Mical potentiates tau toxicity

**Fig.2:**
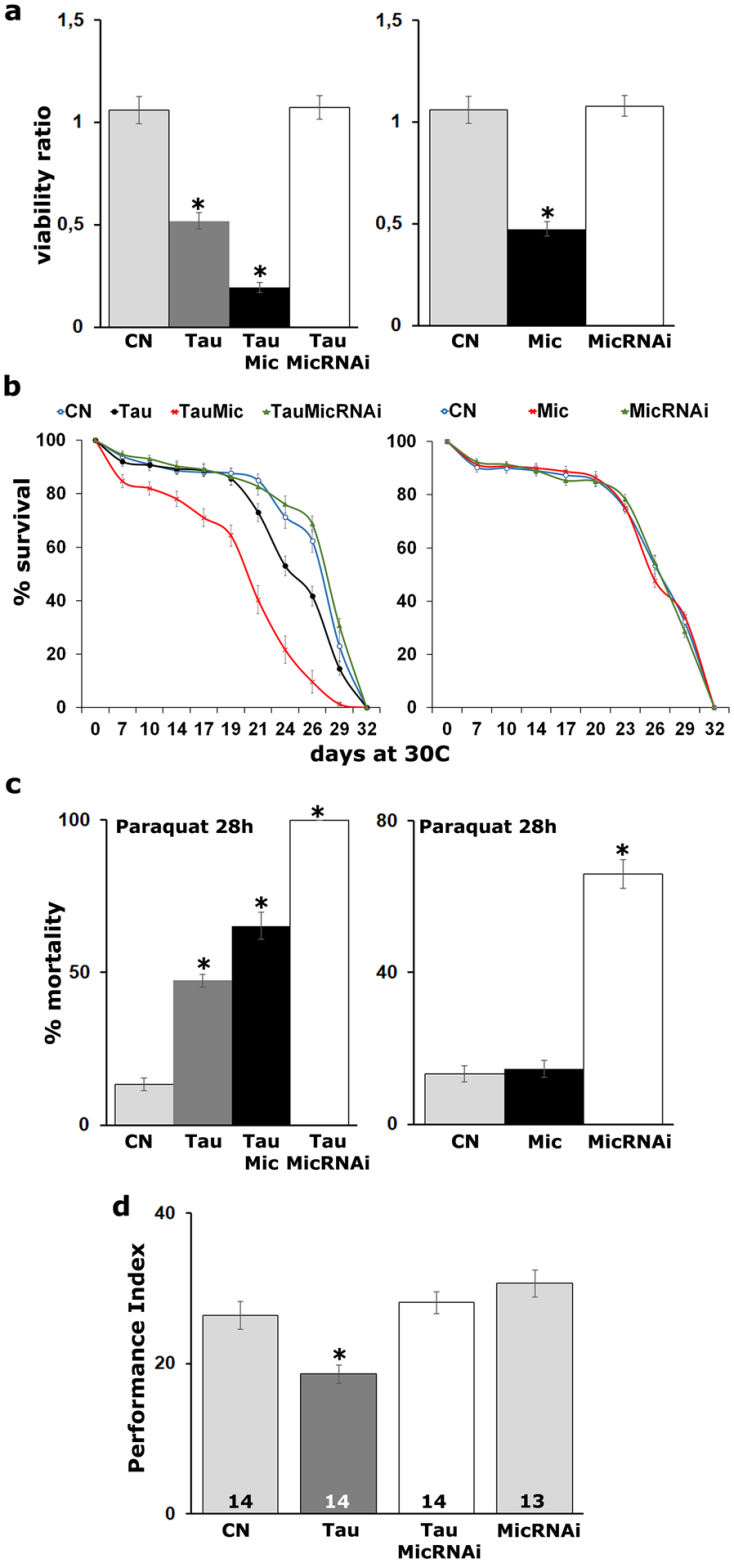
Mical affects Tau toxicity in vivo. **a** Virgin female flies bearing the hTau^0N4R^ transgene were crossed with *elav*^*C155*^*-GAL4*, *elav*^*C155*^*-GAL4*;UAS-Mic/CyO and *elav*^*C155*^*-GAL4*;UAS-MicRNAi/CyO males. Bars represent the mean number of non-balancer-bearing progeny females over males ± SEM of the indicated genotypes. *w*^*1118*^ females were crossed with *elav*^*C155*^*-GAL4* males and the ratio of their progeny female *versus* male was considered as control progeny (CN). *w*^*1118*^ females have equally been crossed with e*lav*^*C155*^*-GAL4*;UAS-Mic/CyO *and elav*^*C155*^*-GAL4*;UAS-MicRNAi/CyO males to assess the viability of Mical transgenes in the absence of Tau (right panel). Stars indicate significant difference from CN. **b** Survival curves for animals expressing panneuronally the indicated transgenes at 30 °C, in comparison with *elavC15-GAL4*/+*;tub-Gal80ts*/+ control (CN). Statistical analysis indicated significant differences in longevity after accumulation of hTau^0N4R^ alone and upon co-overexpression with Mical. **c.** Response of flies expressing panneuronally the indicated transgenes upon treatment with 30 mM paraquat for 28 h. Stars indicate significant difference from control (CN, *elav*^*C155*^*-GAL4*/+). **d** Memory performance of animals expressing in the adult CNS for 12 days hTau^0N4R^ alone and upon attenuation of Mical levels. Controls (light grey bars) were the *elav*^*C155*^*-GAL4*/+*;tub-Gal80ts*/+ flies and animals expressing the UAS-Mical RNAi transgene alone. The genotypes of all animals are indicated below each bar. Star indicates significant differences from both controls. The number of experimental replicates (n) is indicated within the bars

To further explore the interaction between Tau and Mical, we determined whether Mical loss or elevation could indeed affect Tau toxicity. Reduced viability was used as a first read-out and scored as the number of flies that reach adulthood [39, 52]. As already reported [39], panneuronal overexpression of Tau precipitated 50% lethality (Fig. 2a, Tau *vs* control p = 1.7703e−06, n = 6), but its co-expression with excess Mical increased lethality to nearly 80% (Fig. 2a, Tau *vs* TauMical p = 0.0001, n = 6). Even though excess Mical appears toxic on its own (Fig. 2a, Mical *vs* control p = 8.9567e−07, n = 6), its elevation potentiates Tau toxicity. Interestingly, attenuation of Mical levels did not precipitate significant lethality on its own (Fig. 2a, MicRNAi *vs* control p = 0.9555, n = 6) but increased the viability of Tau expressing animals to control levels (Fig. 2a, TauMicRNAi *vs* control p = 0.9931, n = 6).

We next analyzed the effect of Mical levels on the lifespan of Tau-expressing flies (Fig. 2b). To that end, animals were raised at 18 °C to minimize transgene expression during development and then transferred and maintained at 29 °C starting 2 days post-eclosion until they expired. As already reported, Tau expression results in premature lethality relative to controls [40] and animals begin to expire around day 21 (Fig. 2b, Tau day 21 prob ChiSq = 0.0109 through day 29 prob ChiSq < 0.004) with 50% attrition occurring at day 24. Importantly, excess Mical resulted in enhanced mortality starting at day 7 (Fig. 2b, TauMical day 7 prob ChiSq = 0.0121 through day 29 prob ChiSq < 0.001) with 50% attrition occurring at day 20. In contrast, attenuation of Mical levels completely eliminated the reduced longevity phenotype since the life span of these animals was statistically indistinguishable from that of controls (Fig. 2b, TauMicRNAi prob ChiSq > 0.3). Notably, no significant lethality was observed when Mical levels were altered in the absence of transgenic human Tau (Fig. 2b, right panel prob ChiSq > 0.2). Because sex is a major determinant of lifespan [53], we performed an independent lifespan experiment using only male flies and as shown in Additional file 3: Fig. S2 inter-genotype differences were preserved.

A different measure of toxicity that underlies the level of oxidative stress upon pathological Tau accumulation is resistance to exogenous Reactive Oxygen Species inducers such as paraquat [7, 54]. Compared with flies expressing Tau alone, concomitant Mical elevation resulted in significantly higher mortality upon treatment with 30 mM paraquat for 28 h (Fig. 2c, Tau *vs* TauMical p = 0.0002). In contrast, there was no significant lethality upon Mical elevation alone (Fig. 2c, right panel Mical *vs* control p = 0.9784). However, Mical attenuation alone resulted in dramatically enhanced susceptibility to oxidative injury (Fig. 2c, right panel MicRNAi *vs* control p = 9.8861e−09), which most likely underlies the decreased oxidative stress resistance of Tau-expressing flies upon Mical reduction (Fig. 2c, left panel, Tau *vs* TauMicRNAi p = 1.5814e−08).

Collectively, these results indicate that Mical attenuation, apart from resistance to oxidative stress, is sufficient to alleviate Tau toxicity in vivo and that prompted us to determine whether Mical down-regulation could impact Tau-mediated neuronal dysfunction manifested as memory deficits in Tau-expressing animals. As already published [43], panneuronal Tau accumulation during adulthood decreases memory relative to that of controls (Fig. 2d, ANOVA: F_(3,54)_ = 10.8565, p = 1.2604e−05; subsequent LSM: p = 0.00089 *vs* control). Significantly, attenuation of Mical levels eliminated the memory deficit of Tau-expressing animals to control levels (Fig. 2d, ANOVA: F_(3,54)_ = 10.8565, p = 1.2604e−05; subsequent LSM: p = 0.4387 and p = 0.2639 TauMicRNAi *vs* control and MicRNAi respectively). Importantly, abrogation of Mical levels on its own did not affect memory (Fig. 2d, ANOVA: F_(3,54)_ = 10.8565, p = 1.2604e−05; subsequent LSM: p = 0.0637 *vs* control). These results were confirmed with an independent Mical RNAi-encoding transgene (Mical RNAi2, Additional file 4: Fig. S3). Both RNAis effectively reduced Mical levels (Additional file 4: Fig. S3a, RNAi p = 4.07e−06 and RNAi2 p = 1.56e−06, n = 3) and elicited the same behavioral and biochemical effects (Additional file 4: Fig. S3b–e).

Therefore, Mical levels impact not only Tau-mediated toxicity but also Tau-associated neuronal dysfunction, interestingly though without affecting its phosphorylation levels in contrast to its solubility which is highly altered. A potential mechanism underlying these Mical effects could be an oxidation dependent post-translational modification that results in conformational changes independent of the phosphorylation status of Tau.

### Mical’s redox activity mediates the effects on Tau

As Mical bears the characteristic redox enzymatic domain, we wondered whether this activity is necessary to modulate Tau toxicity and interaction with the cytoskeleton. To that end, we used a mutated Mical transgene (MicalΔredox) harboring three amino acid substitutions of tryptophan (W) for glycine (G). These disrupt the dinucleotide binding motif (GxGxxG) of Mical and consequently its redox activity [32]. As already reported [32], the mutated Mical was expressed at levels comparable to those of the wt transgene and both UAS constructs induced a potent increase in Mical levels (Additional file 5: Fig. S4, Δredox p = 4.51e−04, Mic p = 2.36e−05, n = 3).

**Fig. 3:**
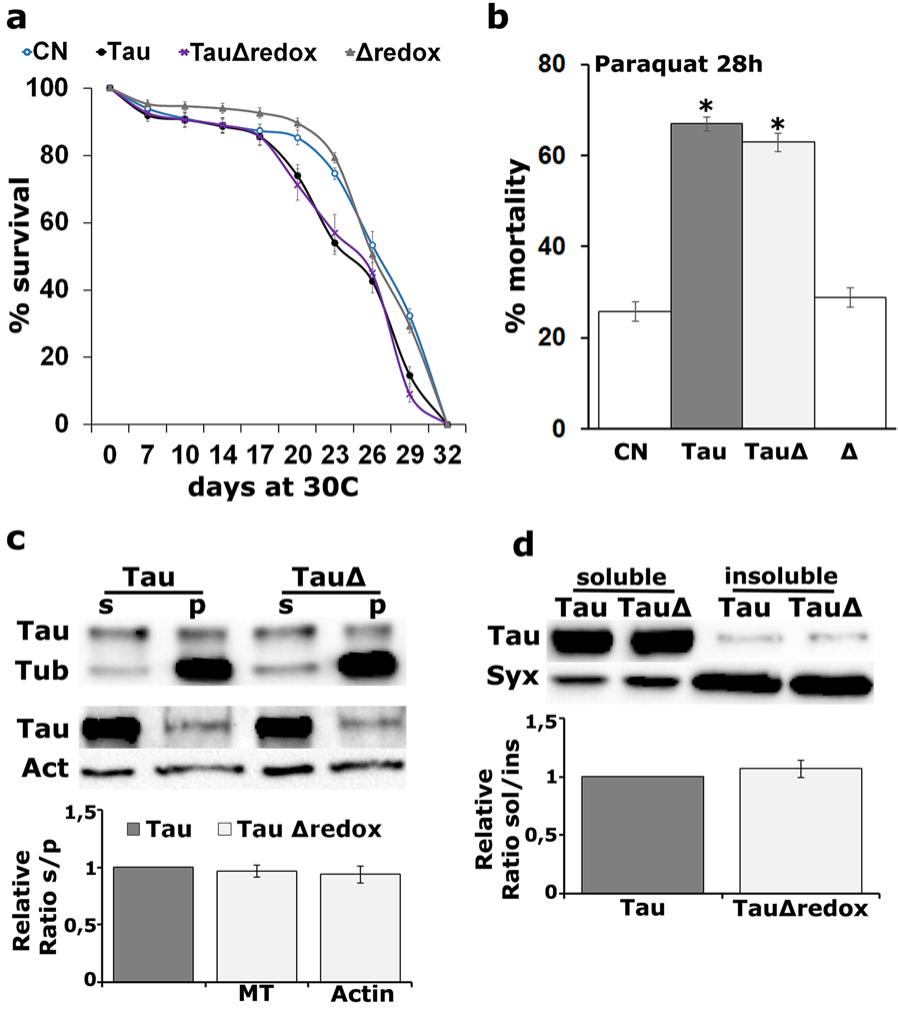
Mical affects Tau via its redox enzymatic activity. **a** Survival curves for animals expressing panneuronally the indicated transgenes at 30 °C, in comparison with *elav*^*C155*^*-GAL4*/+*;tub-Gal80ts*/+ control (CN). Statistical analysis indicated significant differences in longevity after accumulation of hTau^0N4R^ alone and upon co-overexpression with UAS-MicalΔredox mutant transgene. **b** Response of flies expressing panneuronally the indicated transgenes to the oxidant molecule paraquat. Flies have been treated with 30 mM paraquat and mortality was scored after 28 h. Control flies are driver single copy flies (CN, *elav*^*C155*^*-GAL4*/+) and flies that overexpress MicalΔredox. **c** Endogenous microtubules (MT) and phalloidin-bound F-Actin were isolated from flies expressing panneuronally the indicated transgenes. Pellet (p) and supernatant (s) fractions have been probed for Tau, Tubulin and Actin respectively. The ratio of the relative level of Tau in the supernatant to the Tau in the pellet fractions was used for quantification and shown as ratios of their means ± SEM relative to the ratio of Tau expressed alone, which is arbitrarily set to 1. **d** Representative Western blot of aqueous soluble and insoluble fractions generated from adult heads following panneuronal expression of the indicated transgenes. Syntaxin (Syx) was used as loading control. The bars represent the mean ± SEM relative sol/ins ratios of Tau upon MicalΔredox co-overexpression, over that of Tau alone

First, we measured the effects of MicalΔredox expression on lifespan, by co-expressing it with Tau under the control of the conditional panneuronal *elav*^*C155*^*-GAL4; tub-Gal80ts* driver. As shown in Fig. 3a, MicalΔredox does not affect survival of Tau-expressing flies and both genotypes start to expire around day 21 with 50% attrition occurring at day 24 (Fig. 3a, day 21 prob ChiSq = 0.0282 and through day 29 prob ChiSq < 0.02). Survival of MicalΔredox expressing animals was statistically indistinguishable from that of control flies (Fig. 3a, prob ChiSq > 0.07). Similarly, upon administration of 30 mM paraquat, Tau expressing flies presented comparable susceptibility to oxidative injury with flies co-expressing the redox defective Mical (Fig. 3b, Tau *vs* TauMicΔredox p = 0.2825).

Is the redox activity of Mical necessary for the interaction of Tau with the cytoskeleton? Significantly, in contrast to wt Mical, expression of the mutant protein did not alter Tau binding to microtubules or to F-Actin (Fig. 3c, MT p = 0.3310 and Actin p = 0.4350, n = 3). Finally, as shown in Fig. 3d, upon MicalΔredox co-expression with Tau the ratio of soluble/insoluble fraction of the latter remained unaltered (p = 0.3780, n = 4).

To independently confirm these findings, we employed pharmacological inhibition of Mical to ask whether it recapitulates the results of the genetic studies and inhibits Tau aggregation in vivo. Green tea polyphenol ( −)-epigallocatechin gallate (EGCG) is an inhibitor that inactivates the MICAL monooxynase enzymatic activity [55]. Interestingly, EGCG has been shown to inhibit Tau aggregation in vitro [56], to provide cognitive benefits to AD transgenic mice [57] and currently under clinical trial as AD treatment [58]. Mechanistic studies of its neuroprotective effects revealed that EGCG can influence numerous processes acting mostly as an antioxidant [59].

**Fig. 4:**
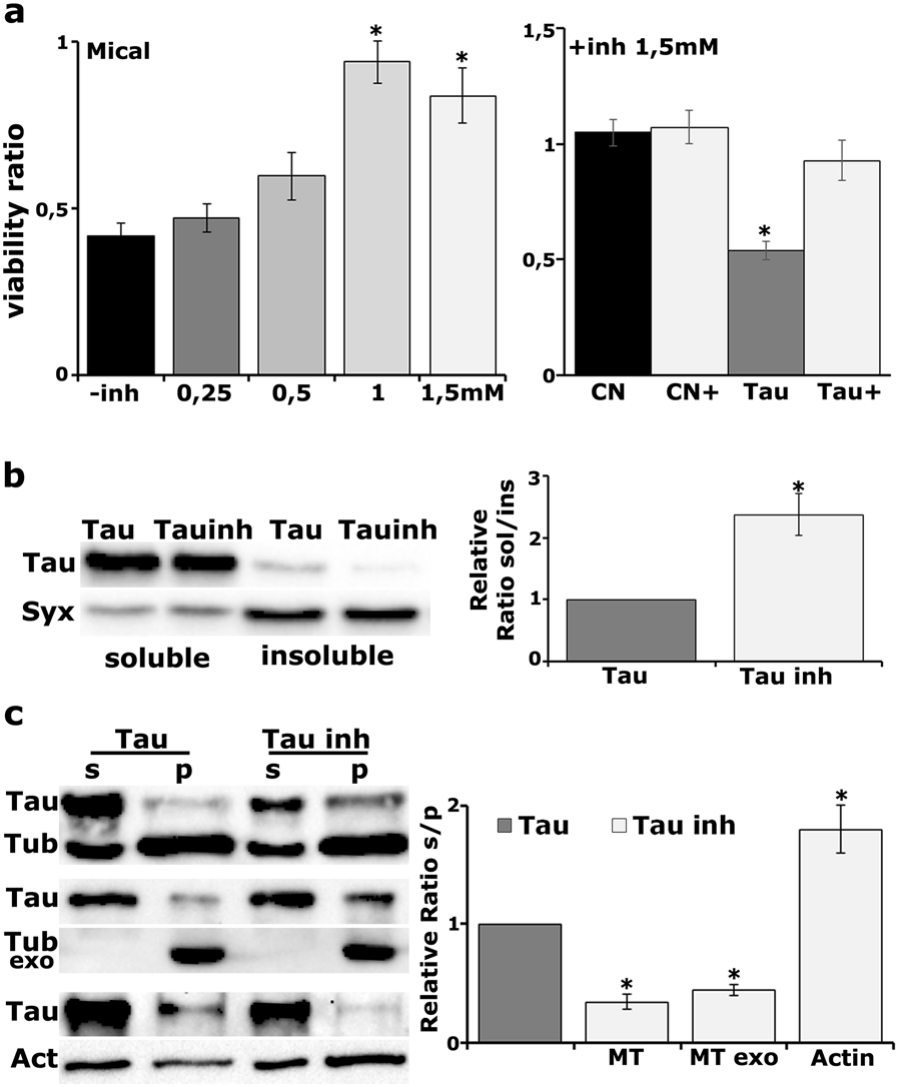
Mical inhibition upon treatment with EGCG. **a** (left panel) Virgin female flies bearing the UAS-Mical transgene were crossed with *elav*^*C155*^*-GAL4* males in a medium supplemented with the indicated concentrations of EGCG. Bars represent the mean ratio number of their progeny female *versus* male ± SEM. Stars indicate significantly altered viability ratios compared to untreated flies (right panel). Virgin female flies carrying the htau^0N4R^ transgene were crossed with *elav*^*C155*^*-GAL4* males in a medium supplemented or not with 1.5 mM EGCG. *w*^*1118*^ females were crossed with *elav*^*C155*^*-GAL4* males and the ratio of their progeny female *versus* male was considered as control progeny without (CN) or with EGCG (CN+). Star indicates significant difference from CN. **b** Representative Western blot of aqueous soluble and insoluble fractions generated from adult heads following panneuronal expression of Tau in the absence or presence of 1.5 mM EGCG. Star indicates significantly increased solubility of Tau upon treatment with EGCG. **c** Pellet (p) and supernatant (s) fractions of endogenous microtubules (MT), preformed bovine microtubules (MTexo) and phalloidin-bound F-Actin (Actin) from Tau expressing flies in the absence or presence of 1.5 mM EGCG have been probed for Tau, Tubulin and Actin respectively. Stars indicate significantly altered levels of Tau co-precipitated with microtubules and Actin upon treatment with 1.5 mM EGCG compared to untreated Tau flies

Determination of the number of adults that emerged upon treatment with EGCG, revealed that the inhibitor restored the viability of Mical overexpressing flies in a dose-dependent manner (Fig. 4a, − inh *vs *+ inh, 0.25 mM p = 0.9610, 0.5 mM p = 0.224, 1 mM p = 0.0004, 1.5 mM p = 7.6747e−05, n = 7). Moreover, treatment with 1.5 mM EGCG restored survival of Tau-expressing flies to control levels (Fig. 4a, Tau *vs* control p = 4.2899e−05, Tau + inh *vs* control p = 0.4475, control *vs* control + inh p = 0.9897, n = 7) and resulted in reduced aggregate formation (Fig. 4b, Tau vs Tau inh p = 0.0208, n = 4). Reassuringly, EGCG treatment completely altered Tau interactions with the cytoskeleton and phenocopied the effects of Mical abrogation. EGCG enhanced the interaction of Tau with microtubules (Fig. 4c, MT p = 0.0005, MTexo p = 0.0002, n = 3) and attenuated its interaction with F-Actin (Fig. 4c, Actin p = 0.0124, n = 3).

Taken together, these results argue that the redox activity of Mical could oxidize Tau at specific amino acid(s) and modulates its pathogenicity and may even be involved in regulating Tau function. Importantly, Mical-mediated Tau oxidation constitutes a novel in vivo post-translational modification of the latter, potentially linked to pathology. Finally, we have potentially revealed a novel mechanism of EGCG’s beneficial action on Tau-associated pathology via Mical inhibition.

### Mical functionally regulates Tau via oxidation of its Cys322

Because of the established role of Tau cysteines on its microtubule and F-Actin binding affinity, aggregation propensity, toxicity and dysfunction [7] we hypothesized that they are the most likely candidates to be modified by Mical-mediated redox activity.

**Fig. 5:**
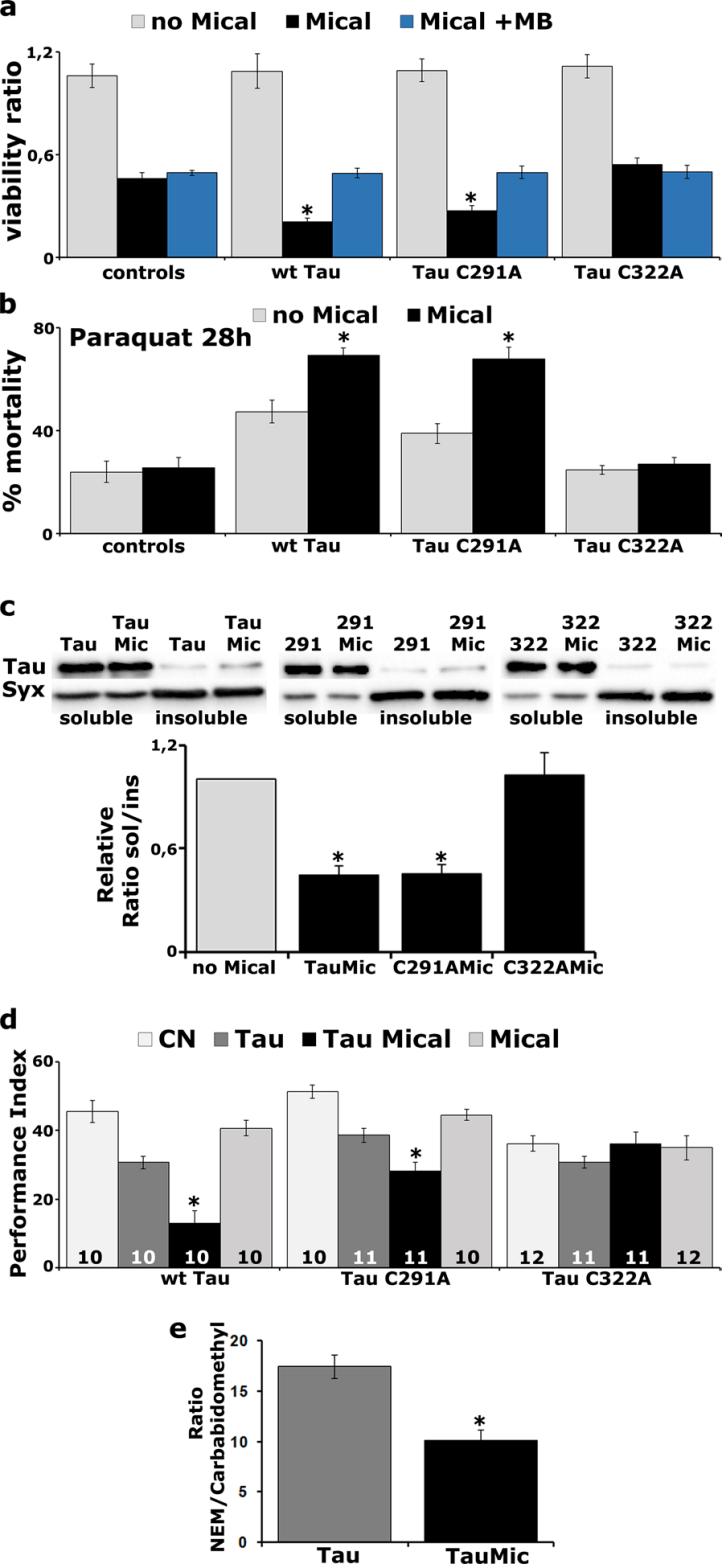
The effects of Mical on Tau are mediated by oxidation of Cys322. **a** Virgin female flies carrying the indicated Tau transgenes were crossed with *elav*^*C155*^*-GAL4* (light grey bar) or *elav*^*C155*^*-GAL4*;UAS-Mical/CyO males in a medium supplemented (blue bar) or not (black bar) with 10 μM Methylene Blue (MB). Controls include *w*^*1118*^ females crossed with *elav*^*C155*^*-GAL4* males (light grey bar) *or elav*^*C155*^*-GAL4*;UAS-Mical/CyO in normal food (black bar) or with MB (blue bar). Bars represent the mean number of non-balancer-bearing progeny females over males ± SEM of the indicated genotypes. Stars indicate significant difference between black and blue bars. **b** Response of flies expressing the indicated transgenes upon treatment with paraquat for 28 h. Stars indicate significant difference from the transgene without Mical overexpression. Control flies are *elav*^*C155*^*-GAL4*/+*;Ras2-GAL4*/+ (grey bar) and Mical are flies that overexpress Mical under the panneuronal double driver (black bar). **c** Representative Western blots of aqueous soluble and insoluble fractions of the indicated Tau transgenes alone or upon co-overexpression with Mical. Stars indicate significantly reduced solubility from the transgene without Mical overexpression. **d** Memory performance of animals expressing panneuronally the indicated Tau transgenes (dark grey bar), compared with the same transgene upon co-expression with Mical (black bars). Stars indicate significant differences from the transgene without Mical overexpression. Control flies (light grey bars) are *elav*^*C155*^*-GAL4*/+*;Ras2-GAL4*/+ flies (CN) and flies that overexpress Mical under the panneuronal double driver. **e** The normalized peak areas were calculated for each of the two variants of the cysteine containing peptide ^322^CGSLGNIHHKPGGGQVEVK and their ratio (NEM *versus* carbamidomethyl) is shown. The bars represent the mean ± SEM ratio from three biological and two technical replicas. Star indicates significant difference between the two groups

To test this hypothesis, we used the viability assay and treated flies with Methylene Blue (MB), a well-known inhibitor of fibrillization that modifies the cysteine residues to sulfenic, sulfinic and sulfonic acid converting Tau to an aggregation incompetent monomeric state [60]. In addition, we used Tau transgenes bearing mutations of each of the two cysteines to alanine (C291A and C322A). To facilitate comparisons, wild-type (wt) hTau^2N4R^ and C291A and C322A mutant transgenes were integrated into the same attp landing site in the fly genome. These transgenes are expressed at lower levels compared to the randomly inserted hTau^0N4R^ construct used until now in this study [27] and as a result, their pan-neuronal expression under the *elav*^*C155*^*-GAL4* driver does not result in reduced viability (Fig. 5a, wt Tau, C291A, C322A *vs* non-transgenic control, p > 0.5, n = 7).

However, Mical overexpression yields 50% lethality and since it potentiates Tau toxicity, their co-expression reached 80% lethality (Fig. 5a, TauMical *vs* Mical, p = 0.00070, n = 7). Interestingly, this effect was suppressed by MB treatment (Fig. 5a, TauMical vs TauMical + MB, p = 0.00018). MB did not affect the viability of Mical expressing flies (Mical vs Mical + MB, p = 0.6542, n = 7) and lethality of MB-treated Tau-Mical co-expressing flies reached the levels of Mical expressing flies alone (Fig. 5a, TauMical + MB *vs* Mical, p = 0.6900, n = 7). Importantly, excess Mical resulted in significant reduction in the viability of the C291A mutant flies (Fig. 5a, C291AMical vs Mical, p = 0.0109, n = 7) but not of the C322A mutant (Fig. 5a, C322AMical vs Mical, p = 0.2467, n = 7). Finally, as with wt Tau, lethality of MB treated C291A mutants was not augmented by Mical excess (Fig. 5a, C291AMical + MB vs Mical, p = 0.6456, n = 7). Therefore, the effects of Mical on Tau are mediated by cysteine oxidation but clearly the two residues are not functionally equivalent.

To further support this notion and determine whether Mical exhibits preferential functional interactions with the Cys291 *versus* Cys322, we examined their survival under conditions of oxidative stress. Mical over-expression enhanced the mortality of Tau-expressing flies exposed to paraquat (Fig. 5b, Tau *vs* TauMical, p = 2.3682e−05). As with wt Tau, flies expressing the C291A mutant challenged with paraquat also presented significantly higher mortality upon Mical excess (Fig. 5b, C291A *vs* C291AMical, p = 1.3597e−07). In contrast, Mical overexpression did not affect paraquat-induced toxicity of flies expressing the C322A mutant (Fig. 5b, C322A *vs* C322AMical, p = 0.6467). We also assessed the solubility profile of these mutant Tau proteins and observed that Mical elevation did not affect the solubility of the C322A mutant, whereas the ratio of soluble/insoluble fraction of wt Tau and the C291A mutant was greatly decreased upon Mical excess (Fig. 5c, Tau *vs* TauMical, p = 6.7716e−06, C291A *vs* C291AMical, p = 7.244e–05, C322A *vs* C322AMical, p = 0.8550, n = 4).

We have previously shown that these Cys to Ala mutations ameliorate the effects of Tau on neuronal dysfunction as flies expressing these mutant proteins are not memory deficient [7]. In addition, Mical overexpression affected Tau interactions with proteins involved in synaptic transmission (Additional file 1: Table S4). Therefore, we hypothesized that Mical overexpression may in fact affect differentially associative memory of wt and the cysteine Tau mutant-expressing animals.

As shown, in Fig. 5d, wt Tau expression resulted in memory deficient flies and co-expression with Mical exacerbated the deficit (Fig. 5d, ANOVA: F_(3,39)_ = 28.4934, p = 1.2874e−09; subsequent LSM: p = 0.00004 Tau *vs* TauMical). It should be noted that Mical excess alone did not affect memory (Fig. 5d, ANOVA: F_(3,39)_ = 28.4934, p = 1.2874e−09; subsequent LSM: p = 0.2056 *vs* control). Significantly however, excess Mical attenuated memory in flies expressing the C291A Tau mutant (Fig. 5d, ANOVA: F_(3,41)_ = 20.2408, p = 5.2888e−08; subsequent LSM: p = 0.001 C291A *vs* C291AMical), but not of animals expressing the C322A mutation (Fig. 5d, ANOVA: F_(3,45)_ = 1.3712, p = 0.2647; subsequent LSM: p = 0.0980 C322A *vs* C322AMical). Therefore, the Mical-regulated oxidation state of Cys322 is critical for Tau mediated neuronal dysfunction.

We have previously reported that mutation of Cys322, but not of Cys291, reduces the expression levels of the transgene compared to wt Tau [7], which could account for the differential effect of Mical on the toxicity of the mutants. To test this hypothesis, we sought to increase the levels of C322A by driving its expression with the double *elav*^*C155*^*-GAL4*;*Ras2-GAL4* driver whereas C291A was expressed under *elav*^*C155*^*-GAL4*. Despite increased expression of C322A under the double panneuronal driver (Additional file 7: Fig. S6a, p = 0.0002), excess Mical did not result in significant viability reduction of the mutant flies (Additional file 7: Fig. S6b, C322AMical vs Mical, p = 0.3840, n = 7). We also challenged flies expressing the C291A mutant with paraquat and observed significantly higher mortality upon Mical excess (Additional file 7: Fig. S6c, C291A *vs* C291AMical, p = 0.0042), despite its lower expression compared to C322A in Fig. 5b. Finally, we tested the memory performance of animals accumulating the C291A mutant under *elav-GAL4* and observed a similar impairment upon Mical excess as in Fig. 5d (Additional file 7: Fig. S6d, ANOVA: F_(3,39)_ = 6.1694, p = 0.0017; subsequent LSM: p = 0.0023 C291A *vs* C291AMical). Therefore, the observed phenotypic differences among the mutant Tau proteins are not due to differences in expression, but rather reflect differential functional effects.

Initial in vivo evidence that Tau forms disulfide bonds in the fly retina was provided by Saito et al. [61], so to quantify oxidized cysteine residues upon Mical overexpression in the fly CNS, we utilized a targeted proteomics approach. Briefly, N-ethyl maleimide (NEM) is known to react with free-thiols resulting in maleimide adducts that are readily observed in the MS spectra as a 125 Da shift in molecular mass. Then a reduction of pre-oxidized cysteines via DTT treatment was performed followed by a second alkylation step with iodoacetamide, where newly formed sulfhydryl groups were capped with carbamidomethyl groups (+ 57 Da) [62]. Subsequent analysis by liquid chromatography-tandem mass spectrometry (Additional file 6: Fig. S5a, b) revealed that the ratio of Cys322 containing peptide (^**322**^**C**GSLGNIHHKPGGGQVEVK) modified by NEM *versus* the modified by the carbamidomethyl counterpart was greatly reduced upon Mical overexpression (Fig. 5e, Tau *vs* TauMical, p = 0.0027) indicating increased cysteine oxidation. It should be noted, that peptides containing the Cys291 residue were not captured by the column and therefore not detected in this proteomic analysis. Collectively the results clearly indicate that the effects of Mical on Tau-mediated toxicity and dysfunction are mediated by Cys322 oxidation.

### Mical levels also impact the toxicity and dysfunction of 3R Tau isoforms

Based on the above results and if our conclusion is correct, then altering Mical levels should also functionally impact animals expressing the 3R tau isoform that contains Cys322, but not Cys291.

**Fig. 6:**
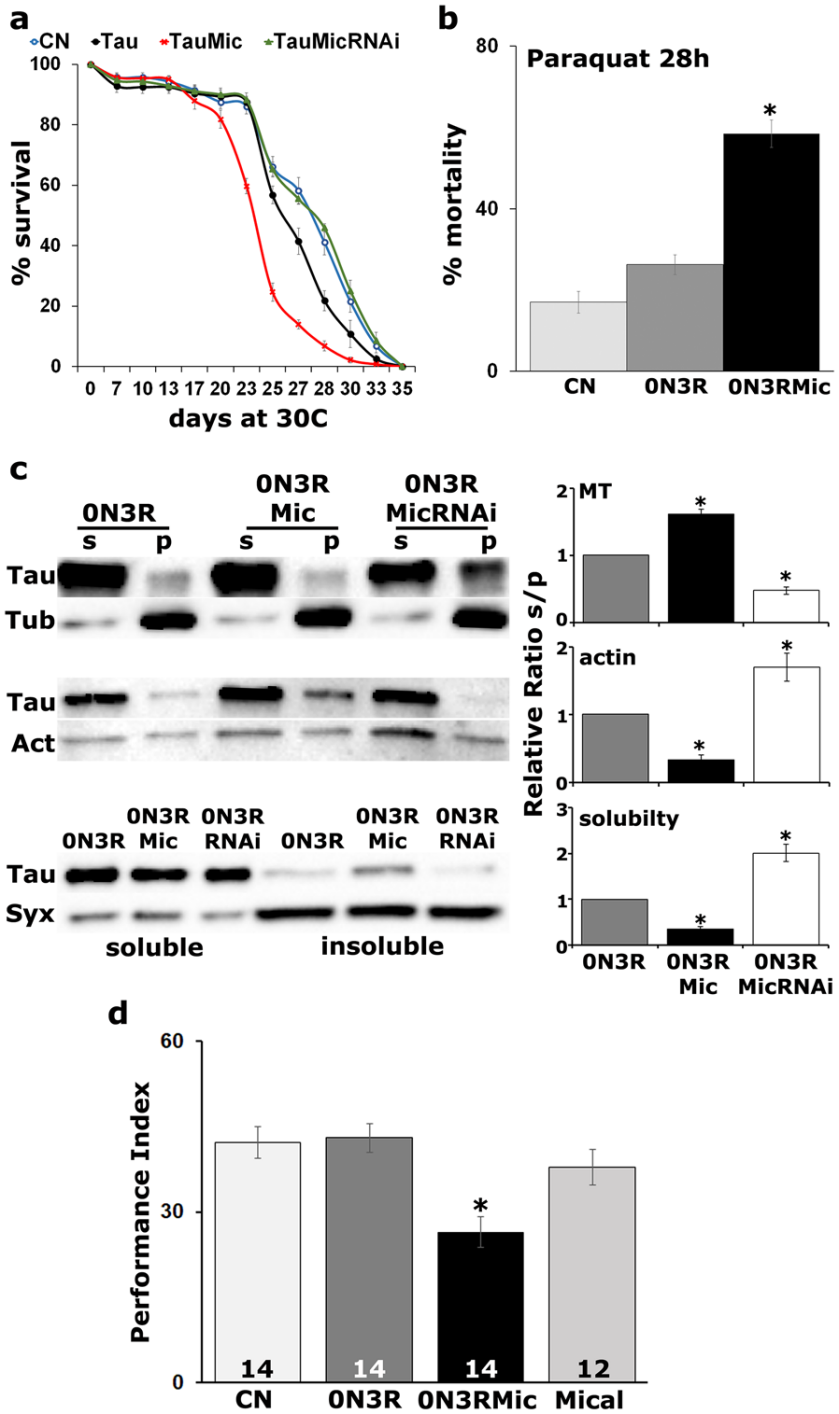
Mical equally affects the neurotoxicity of hTau^0N3R^ isoform. **a** Survival curves for animals expressing panneuronally the indicated transgenes at 30 °C, in comparison with *elav*^*C155*^*-GAL4*/+*;tub-Gal80ts*/+ controls (CN). Statistical analysis using the log rank test indicated significant differences in longevity after accumulation of hTau^0N3R^ alone and upon co-overexpression with Mical. **b** Response of flies expressing panneuronally the hTau^0N3R^ transgene (dark grey bar) to the oxidant molecule paraquat, compared with the same transgene upon co-overexpression of Mical (black bar). Star indicates significant difference from the transgene without Mical overexpression. Control flies (CN) are driver elav^C155^-GAL4/+ flies (light grey bar). **c** Endogenous microtubules (upper panel), phalloidin-bound F-Actin (middle panel) and aqueous soluble and insoluble fractions (lower panel) were isolated from flies expressing under the *elav*^*C155*^*-GAL4* driver the hTau^0N3R^ transgene alone or upon Mical up and down-regulation. p: pellet and s: supernatant fractions were analyzed by western blotting using antibodies against Tau (5A6), Tubulin (E7), Actin and Syntaxin. Stars indicate significantly altered levels of precipitated 0N3R upon modulation of Mical levels compared to Tau expressed alone. **d** Memory performance of animals expressing panneuronally the hTau^0N3R^ transgene (dark grey bar), compared with the same transgene upon co-expression with Mical (black bar). Control flies (light grey bars) are driver *elav*^*C155*^*-GAL4/*+ flies (CN) and flies that overexpress Mical under the panneuronal *elav*^*C155*^*-GAL4* driver. Star indicates significant differences from both controls

Indeed, Mical overexpression significantly shortened the lifespan of hTau^0N3R^ expressing flies (Fig. 6a, TauMical after day 20 prob ChiSq < 0.0001 whereas Tau day 26 prob ChiSq = 0.0309 and through day 29 prob ChiSq < 0.05). Conversely, co-expression of hTau^0N3R^ with a Mical-abrogating RNAi transgene extended their lifespan to control levels (Fig. 6a, TauMicRNAi prob ChiSq > 0.5). Moreover, hTau^0N3R^ transgenic flies appeared more vulnerable to paraquat than control flies (Fig. 6b, 0N3R *vs* control p = 0.0514) and Mical overexpression further hypersensitized them to oxidative injury (Fig. 6b, 0N3R *vs* 0N3RMical p = 3.6125e−10).

In addition, as shown in Fig. 6c, attenuation of Mical levels strengthened the interaction of hTau^0N3R^ with microtubules, decreased its affinity for F-Actin and increased its solubility (MT p = 0.0007, Actin p = 0.0122, solubility p = 0.0015, n = 3). In contrast, Mical excess precipitated the opposite effects (MT p = 0.0003, Actin p = 0.0150, solubility p = 0.0131, n = 3). We finally addressed the effect of Mical excess on the memory of hTau^0N3R^ -expressing animals. Panneuronal accumulation of the 3R isoform did not affect memory (Fig. 6d, ANOVA: F_(3,53)_ = 7.8466, p = 0.0002; subsequent LSM: p = 0.8360 0N3R *vs* control), as already reported before [31, 43, 63]. In contrast, excess Mical along with Tau resulted in significant memory impairment (Fig. 6d, ANOVA: F_(3,53)_ = 7.8466, p = 0.0002; subsequent LSM: p = 0.00015 and p = 0.0067 0N3RMical *vs* control and Mical transgene respectively). Altogether, these results suggest that the interaction between Mical and Tau is isoform independent and redox-state modulation of the single Cys322 residue is sufficient to regulate hTau neurotoxicity and dysfunction in vivo.

### MICAL1 is upregulated in Tauopathy patients

The novel effects of Mical on Tau-mediated toxicity and dysfunction in the fly Tauopathy model suggested that the levels or localization of the human Mical orthologue MICAL1 may in fact be altered in Tauopathy patients compared to non-demented controls. To address this hypothesis, samples from Alzheimer’ disease (AD), Pick’s disease (PiD) and Frontotemporal Dementia (FTD) patients with already reported [40] detailed description of their neuropathological evaluation were used.

**Fig. 7:**
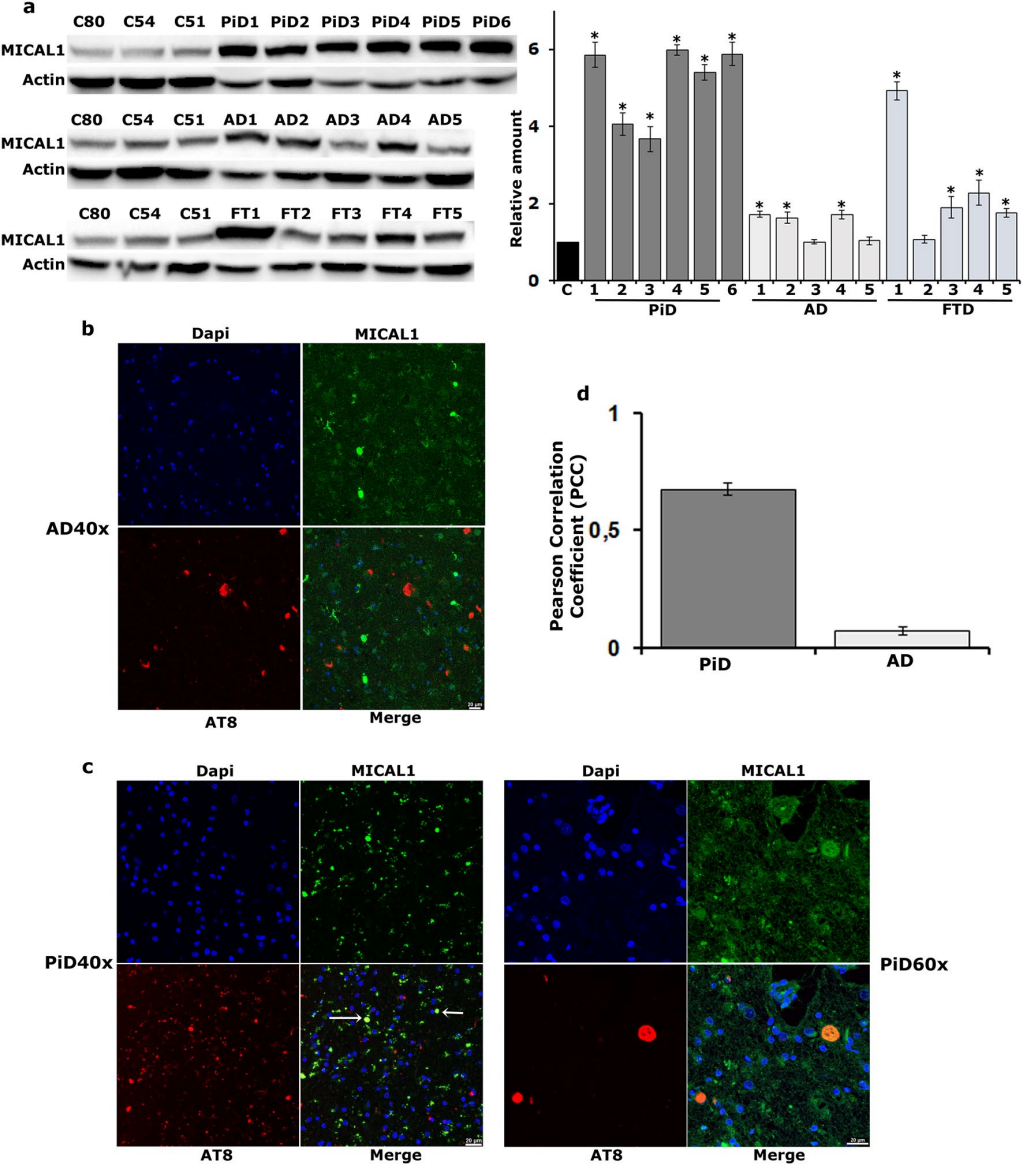
MICAL1 is upregulated in the brain of Tauopathy patients. **a** Western blots of homogenates from hippocampal samples of six Pick’s disease (PiD), five Alzheimer’s disease (AD) and five Frontotemporal Dementia (FT) patients as well as three control subjects (C) whose age is indicated by the respective numbers. Blots were probed for MICAL1 and Actin. Quantifications are shown on the right in which levels of the control samples were normalized using the Actin loading control and their ratio was fixed to 1. The bars represent the mean ± SEM relative levels of MICAL1 at the given hippocampal sample over that of MICAL1 in controls. Stars indicate significantly increased MICAL1 levels in disease patients. **b** Representative sections from the hippocampi of patient subjects with AD at 40 × magnification stained for Tau (AT8 antibody) and MICAL1. The merged sections indicate no colocalization between MICAL1 and Tau in flame-shaped neurofibrillary tangles. **c** Representative sections from the hippocampi of patient subjects with PiD at 40 × and 60 × magnification stained for Tau (AT8 antibody) and MICAL1. White arrows indicate globular Pick bodies. The merged sections indicate prominent colocalization between MICAL1 and Tau in spherical Pick bodies. **d** Mean PCC values ± SEM indicate high degree of colocalization between Tau and MICAL1 in PiD samples (PCC values close to 1) but no colocalization in AD samples (PCC values close to zero)

Significantly and in accord with the hypothesis, Western blots of hippocampal tissue from AD, FTD and PiD subjects revealed that MICAL1 is in fact up-regulated in all 6 PiD subjects compared to non-demented controls (Fig. 7a, p < 7e−06, n = 3 for all samples) and in 3/5 AD (Fig. 7a: AD1 p = 0.0006, AD2 p = 0.001, AD3 p = 0.9, AD4 p = 0.0006 and AD5 p = 0.9, n = 3) and 4/5 FTD (Fig. 7a, FT1 p = 8.6e−12, FT2 p = 0.9, FT3 p = 0.0315, FT4 p = 0.004 and FT5 p = 0.04) subjects. Interestingly, histological evaluation of samples from the same subjects revealed that Tau did not colocalize with MICAL1 in neurofibrillary tangles from AD brains (Fig. 7b), but it did with MICAL1 in Pick bodies (Fig. 7c). Pearson’s correlation coefficient (PCC) was used as a statistic for quantifying colocalization (Fig. 7d). PCC values close to 1, reflecting high degree of colocalization, were observed only for PiD samples. A plausible explanation of this observation is that it reflects the recently published cryo-EM structures of Tau filaments from AD and Pick’s disease [64]. In the Alzheimer fold adopted by all six Tau isoforms, Cys322 is buried within the fibril core whereas in the Pick fold adopted only by 3R isoforms, Cys322 is exposed and located onto a loop thus allowing access to the enzyme. Nevertheless, our results suggest that MICAL1 could serve as a potential biomarker of Tau-related pathology and a specific marker of Pick bodies.

## Discussion

Cys291 and Cys322, the only two cysteine residues of Tau, act as key amino acids in the intrinsic catalytic activity of Tau as acetyltransferase [65], and as residues within the microtubule-binding repeat region are important for the correct localization of Tau on microtubules [66] but also instrumental in the initiation of Tau aggregation [12–14, 67]. We have recently highlighted the importance of cysteines for protein structure stabilization and for specific physiological and pathophysiological functions of Tau, including association with the cytoskeleton, neuronal toxicity and dysfunction in vivo by using the C322A and C291A mutants [7]. Here we explore the effects of the novel Tau interactor Mical, which modulates the redox state of these cysteines acting as a catalyst for aggregation in vivo*.* Apparently, the state of Cys322 oxidation also modulates interactions with a variety of binding partners impacting Tau-associated toxicity and dysfunction in an isoform-independent manner. Intriguingly, elimination of Cys322 significantly suppressed phosphorylation of Tau at disease-associated epitopes [7] whereas the modulation of its redox state had no impact on them (Additional file 2: Fig. S1e). This suggests that cysteine substitution precipitates more significant conformational changes than its redox state, potentially affecting the phosphorylation status of the protein.

It is currently unclear however, but under investigation, whether Mical selectively modifies Cys322, or alters both residues but oxidation of Cys322 acts as a “gatekeeper” of Tau-mediated toxicity and dysfunction. A similar role has been proposed for the phosphorylation of particular sites, thought to promote conformations that also lead to Tau pathogenicity [27, 40]. In that sense, it is interesting that preventing cysteine oxidation does not affect Tau phosphorylation, at least at the sites surveyed (Additional file 2: Fig. S1e), suggesting that the two types of PTMs are independent.

An issue with potentially ameliorative value for Tauopathies is whether the Mical-driven oxidation of Tau cysteines is reversible. Cysteine oxidation may be reversed by the thioredoxin/thioredoxin reductase and glutathione/glutaredoxin couples [68]. Interestingly, Mical excess decreased the interaction between Tau and Thioredoxin reductase-1 (Trxr, Additional file 1: Table S3), whereas downregulation of Trxr antioxidant activities was shown to enhance neurodegeneration in a Drosophila Tauopathy model [54]. Mical excess also affects a number of Tau interactors implicated in cell redox-homeostasis like Peroxiredoxin 5 (Prx5, Additional file 1: Table S3), likely accounting for the decreased longevity [69, 70] and increased susceptibility of Tau-Mical co-overexpressing flies to oxidative stress (Figs. 2b, c, 5b, 6a, b).

Of equal importance are the changes in the interaction with proteins involved in chemical synaptic transmission like acetyl cholinesterase (Ace) and the Vesicular acetylcholine transporter (VAChT) (Additional file 1: Table S4), as well as proteins regulating synaptic plasticity and memory like CamKII and CASK [71], that potentially impact memory performance of Tau-Mical co-overexpressing flies (Figs. 5d and 6d).

However, among the proteins that emerged from the proteomic results (Fig. 1a), an established Tau interactor and modulator of its toxicity, 14–3–3 epsilon [39] provides additional validation for the screen. 14–3–3s are a family of dimeric proteins that bind to serine/threonine-phosphorylated residues in a context specific manner and regulate essential biological processes [72]. A characteristic mode of their action is that they serve as scaffolds to bring two client proteins together. Future work will address the hypothesis that 14–3–3 epsilon bridges Tau and Mical together. Since pathogenic Tau phosphorylation increases potential 14–3–3 binding sites and probably engages additional dimers, we will also investigate whether the interaction is phosphorylation dependent using available phospho-mutants [27, 40].

Increase in Mical levels also leads to decreased interaction of Tau with microtubules and decreased interaction with microtubule-associated proteins such as dTau, Shot and Jupiter (Figs. 1b, 6c and Additional file 1: Table S1). Interestingly, studies in Drosophila models of Tauopathy revealed that the strength of Tau interaction with the MT cytoskeleton correlates with its toxicity. Toxic Tau variants bind very poorly to microtubules and are found mostly as soluble cytosolic hyperphosphorylated forms [73]. Additional studies showed that Actin cytoskeletal changes are equally important mediators of Tau induced neuronal toxicity [74] and components of the Actin cytoskeletal network act as enhancers of neurotoxicity [75, 76]. Interestingly, even though Mical overexpression destabilizes Actin filaments [22], it enhances Tau toxicity. On the other hand, Mical attenuation also affects the interaction of Tau with cytoskeletal proteins (Additional file 1: Table S5), reduces the binding of Tau to F-Actin, abolishes Tau toxicity and even restores the deficient memory of Tau-expressing animals (Fig. 2d).

The importance of the Tau-Mical interaction as a novel molecular mechanism underlying the development of Tau pathology has been reinforced by the finding that human MICAL1 is up-regulated in brain tissue samples from individuals with Tauopathies as compared to non-demented controls (Fig. 7a). Over the past five years, structure determination of Tau filaments from human brain by cryo-electron microscopy (cryo-EM) has provided evidence for the existence of multiple Tau conformers, adopting distinct filament folds between diseases [67]. Interestingly, cryo-EM studies of Tau filaments from human patients revealed that the two cysteine residues are not structurally equivalent since Cys322 is incorporated into the core of the fibril whereas Cys291 is disordered and located in the fuzzy-coat [77]. We hypothesize that these distinctive structural and conformational properties between Neurofibrillary Tangles and Pick bodies could determine Cys322 accessibility and account for the differential immunoreactivity with MICAL1 (Fig. 7b, c) since Mical impacts equally 3R as well as 4R isoforms (Figs. 1, 2, 3, 4, 5, 6). Finally, it should be noted that the examination of AD sections by confocal microscopy reveal positive immunofluorescence for MICAL1 (Fig. 7b), however the nature of this staining i.e. whether it colocalizes with other proteins is currently unknown.

## Conclusions

Our work provided mechanistic insights into the process by which Tau self-assembles into fibrillar inclusions in vivo and improved our knowledge on the role of cysteine oxidation on its function as a cytoskeletal protein and on Tau-associated brain toxicity and dysfunction in vivo. Moreover, our finding that MICAL levels increase in the brain of Tauopathy patients can guide the development of novel biomarkers for clinical diagnostics. Finally, Tau oxidation by MICAL likely defines a critical event in Tau pathogenesis and inhibition of MICAL would in principle offer a new intervention approach.

## Supplementary Information

**Additional file 1.** Proteins interacting differentially with human Tau upon modulation of Mical levels. Selected proteins shown in alphabetical order, p-value and average log2 fold differences from three biological and three to four technical replicas have been calculated as described in Methods. The log2 fold change becomes positive when the affinity for Tau is increased and negative when it is decreased. The t-test was performed with a permutation-based FDR (False Discovery Rate of 0.05) calculation and the p-value determines the statistical significance. **Table S1**. Proteins implicated in microtubule cytoskeleton organization interacting differentially with human Tau upon Mical over-expression. **Table S2**. Proteins implicated in Actin cytoskeleton organization interacting differentially with human Tau upon Mical over-expression. **Table S3**. Proteins implicated in oxidation-reduction processes interacting differentially with human Tau upon Mical over-expression. **Table S4**. Proteins implicated in synaptic transmission interacting differentially with human Tau upon Mical over-expression. **Table S5**. Proteins implicated in cytoskeleton organization interacting differentially with human Tau upon Mical down-regulation. Selected proteins implicated in microtubule (upper group) and Actin (lower group) cytoskeleton organization are shown in alphabetical order. 14-3-3 epsilon does not interact differentially with Tau upon attenuation of Mical levels (p-value>0.05). The other proteins are differential Tau interactors when Mical is either up or down-regulated. Proteins in bold present opposite abundances in the two conditions.

**Additional file 2: Fig. S1 a** Verification of the Tau-Mical interaction via immunoblot analysis. Immunoprecipitation of htau^FLAG−2N4R^ using anti-FLAG coated beads and subsequent western blot analysis using an anti-Mical antibody (+ bait). Anti-FLAG coated beads have equally been mixed with a lysate from flies overexpressing Mical in the absence of htau^FLAG−2N4R^ to ensure non-specific binding of Mical to the beads (-bait). **b** Representative Western blots from head lysates of flies expressing Tau panneuronally compared with similar lysates co-expressing UAS-Mical or a UAS-Mical RNAi transgene probed for 14–3-3 epsilon and Syntaxin. The bars represent the mean ± SEM relative levels of 14–3-3 epsilon upon modulation of Mical levels. **c** Representative Western blots from head lysates of flies expressing Tau panneuronally compared with similar lysates co-expressing UAS-Mical or a UAS-Mical RNAi transgene probed for dTau and Syntaxin. The bars represent the mean ± SEM relative levels of dTau upon modulation of Mical levels. **d** Immunoprecipitation of htau^FLAG−2N4R^ using anti-FLAG coated beads and subsequent western blot analysis using an anti-dTau antibody (+ bait). Anti-FLAG coated beads have equally been mixed with a lysate of *elav*^*C155*^*-GAL4*/+*;Ras2-GAL4*/+ flies to ensure non-specific binding of dTau to the beads (-bait). **e** Representative Western blots from head lysates of flies expressing Tau panneuronally compared with similar lysates co-expressing UAS-Mical or a UAS-Mical RNAi transgene probed with the indicated antibodies. Quantifications of four independent biological replicates are shown below in which levels of the phosphorylated protein were normalized using the Syntaxin (Syx) loading control. The normalized level of Tau expressed alone for each quantification was fixed to 1. The bars represent the mean ± SEM relative levels of Tau phosphorylated at the given site upon modulation of Mical levels over that of Tau expressed alone.

**Additional file 3: Fig. S2** Survival curves for male animals expressing panneuronally the indicated transgenes at 30 °C, in comparison with *elav*^*C155*^*-GAL4*/+*;tub-Gal80ts*/+ controls (CN). The data represent the mean ± SEM from two independent experiments with a total of 300 flies assessed per genotype. Statistical analysis indicated significant differences in longevity after accumulation of hTau^0N4R^ alone and upon co-overexpression with Mical. Tau day 21 prob ChiSq = 0.00006 through day 29 prob ChiSq < 0.0002, TauMical day 10 prob ChiSq = 0.0009 through day 29 prob ChiSq < 0.00002 and TauMicRNAi prob ChiSq > 0.2.

**Additional file 4: Fig. S3** Mical down-regulation with a second independent RNAi line. **a** Representative Western blot of head lysates from flies expressing UAS-Mical RNAi lines and probed with anti-Mical antibody. RNAi is line 18668R-2 and RNAi2 is line 25,372. The genotype of control animals was *elav*^*C155*^*-GAL4*/+*.* Stars indicate significant differences from control. **b** Endogenous microtubules (MT), phalloidin-bound F-Actin and aqueous soluble and insoluble fractions were isolated from flies expressing panneuronally the indicated transgenes. Pellet (p) and supernatant (s) fractions have been probed for Tau, Tubulin, Actin and Syntaxin respectively. Stars indicate significantly altered levels of precipitated Tau upon down-regulation of Mical levels (MT p = 0.0007, Actin p = 0.0006, sol p = 9.76e-0.5, n = 3). **c** Virgin female flies bearing the hTau^0N4R^ transgene were crossed with *elav*^*C155*^*-GAL4*;UAS-MicRNAi2/CyO males. *w*^*1118*^ females were crossed with *elav*^*C155*^*-GAL4* males (control progeny CN) and with *elav*^*C155*^*-GAL4*;UAS-MicRNAi2/CyO males to assess the viability of UAS-Mical RNAi2 transgene. Attenuation of Mical levels did not precipitate significant lethality on its own (MicRNAi2 *vs* control p = 0.9992, n = 6) but increased viability of Tau expressing animals to control levels (TauMicRNAi2 *vs* control p = 0.9876, n = 6). **d** Survival curves for animals expressing the indicated transgenes in comparison with *elav*^*C155*^*-GAL4*/+*; tub-Gal80ts*/+ controls. The life span of animals coexpressing hTau^0N4R^ and Mical RNAi2 was statistically indistinguishable from that of controls (TauMicRNAi2 prob ChiSq > 0.3). **e** Response of flies expressing UAS-Mical RNAi2 upon treatment with paraquat for 28 h. Star indicates significant difference from control *elav*^*C155*^*-GAL4*/+ flies (p = 4.6905e−06). **f** Memory performance of animals expressing in the adult CNS for 12 days hTau^0N4R^ alone and upon attenuation of Mical levels. Controls (light grey bars) were the *elav*^*C155*^*-GAL4*/+*;tub-Gal80ts*/+ and animals expressing the UAS-Mical RNAi2 transgene alone. Star indicates significant differences from both controls (ANOVA: F_(3,39)_ = 5.0557, p = 0.0050; subsequent LSM: p = 0.6071 and p = 0.5992 TauMicRNAi *vs* control and MicRNAi respectively).

**Additional file 5: Fig. S4** UAS-Mical and UAS-MicalΔredox transgenes are expressed at comparable levels. Representative Western blot of head lysates from flies expressing the two UAS-Mical transgenic lines using *elav*^*C155*^*-GAL4* and probed with anti-Mical antibody. The genotype of control animals was *elav*^*C155*^*-GAL4*/+*.* Stars indicate significant differences from control (CN).

**Additional file 6: Fig. S5** Targeted proteomics to quantify cysteine oxidation. **a** Extracted chromatograms for the parent ions and isotopes (upper panel) and of its 6 most abundant fragments (daughter ions, lower panel) at the retention time 27.9 min of the NEM and carbamidomethyl labeled ^322^CGSLGNIHHKPGGGQVEVK peptide from representative samples of Tau and Tau co-overexpressed with Mical. **b** Spectra of the scan used for the library creation of the NEM (+ 125 Da) and carbamidomethyl (+ 57 Da) modified ^322^CGSLGNIHHKPGGGQVEVK peptide.

**Additional file 7: Fig. S6 a** Representative Western blot of head lysates from flies expressing UAS-htau^FLAG−2N4RC322A^ using *elav*^*C155*^*-GAL4;Ras2-GAL4* and UAS-htau^FLAG−2N4RC291A^ using *elav*^*C155*^*-GAL4*. Star indicates significant differences between the two groups. **b** Virgin *elav*^*C155*^*-GAL4;Ras2-GAL4* females were crossed with UAS-Mic/CyO and UAS-Mic/CyO;UAS-C322A males. Bars represent the mean number of non-CyO bearing progeny over CyO flies ± SEM of the indicated genotypes. **c** Response of flies expressing UAS-htau^FLAG−2N4RC291A^ upon treatment with paraquat for 28 h. Star indicates significant difference from the transgene without Mical overexpression. Control flies are *elav*^*C155*^*-GAL4*/+ (grey bar) and Mical are flies that overexpress Mical under the panneuronal driver (black bar). **d** Memory performance of animals expressing panneuronally the htau^FLAG−2N4RC291A^ transgene (dark grey bar), compared with the same transgene upon co-expression with Mical (black bar). Star indicates significant difference between the two genotypes. Control flies (light grey bars) are driver *elav*^*C155*^*-GAL4*/+ flies (CN) and flies that overexpress Mical. The number of experimental replicates (n) is indicated within the bars.

## Data Availability

Proteomics datasets used in this study are available from the corresponding author upon reasonable request.

## Abbreviations

AD: Alzheimer’s disease
CNS: Central Nervous System
cryo-EM: Cryo-electron microscopy
DTT: Dithiothreitol
CN: Control
MTexo: Exogenous microtubules
MTendo: Endogenous microtubules
FDR: False Discovery Rate
FA: Formic acid
LFQ: Label Free Quantitation
FTD: Frontotemporal Dementia
EGCG: Green tea polyphenol (−)-epigallocatechin gallate
LSM: Least Squares Means
MT: Microtubules
LC–MS: Liquid chromatography–mass spectrometry
MB: Methylene Blue
Mic: Mical
NBB: Netherlands Brain Bank
NEM: N-ethyl maleimide
PMT: Post-translational modification
redox: Reduction–oxidation
SEM: Standard Error of the Mean
Syx: Syntaxin
wt: Wild-type
