# Correction to: Mical modulates Tau toxicity via cysteine oxidation in vivo

**DOI:** 10.1186/s40478-022-01369-w

**Published:** 2022-04-25

**Authors:** Engie Prifti, Eleni N. Tsakiri, Ergina Vourkou, George Stamatakis, Martina Samiotaki, Efthimios M. C. Skoulakis, Katerina Papanikolopoulou

**Affiliations:** 1 Institute for Fundamental Biomedical Research, Biomedical Sciences Research Centre “Alexander Fleming”, 34 Fleming Street, 16672 Vari, Greece; 2 Institute for Bio-Innovation, Biomedical Sciences Research Centre “Alexander Fleming”, 34 Fleming Street, 16672 Vari, Greece; 3 Laboratory of Genetics, Department of Biotechnology, Agricultural University of Athens, Iera Odos 75, 11855 Athens, Greece

## Correction to: Acta Neuropathologica Communications (2022) 10:44 10.1186/s40478-022-01348-1

Following publication of the original article [1], it was noted that due to a typesetting mistake, incorrect files for Additional files 6 and 7 were processed.

The correct files for Additional files 6 and 7 are attached to this Correction and have been corrected in the original article. The publisher apologises to the authors and readers for the inconvenience caused by this error.

## Supplementary Information

**Additional file 6: Fig. S5** Targeted proteomics to quantify cysteine oxidation. **a** Extracted chromatograms for the parent ions and isotopes (upper panel) and of its 6 most abundant fragments (daughter ions, lower panel) at the retention time 27.9 min of the NEM and carbamidomethyl labeled ^322^CGSLGNIHHKPGGGQVEVK peptide from representative samples of Tau and Tau co-overexpressed with Mical. **b** Spectra of the scan used for the library creation of the NEM (+ 125 Da) and carbamidomethyl (+ 57 Da) modified ^322^CGSLGNIHHKPGGGQVEVK peptide.

**Additional file 7: Fig. S6 a** Representative Western blot of head lysates from flies expressing UAS-htau^FLAG−2N4RC322A^ using *elav*^*C155*^*-GAL4;Ras2-GAL4* and UAS-htau^FLAG−2N4RC291A^ using *elav*^*C155*^*-GAL4*. Star indicates significant differences between the two groups. **b** Virgin *elav*^*C155*^*-GAL4;Ras2-GAL4* females were crossed with UAS-Mic/CyO and UAS-Mic/CyO;UAS-C322A males. Bars represent the mean number of non-CyO bearing progeny over CyO flies ± SEM of the indicated genotypes. **c** Response of flies expressing UAS-htau^FLAG−2N4RC291A^ upon treatment with paraquat for 28 h. Star indicates significant difference from the transgene without Mical overexpression. Control flies are *elav*^*C155*^*-GAL4*/+ (grey bar) and Mical are flies that overexpress Mical under the panneuronal driver (black bar). **d** Memory performance of animals expressing panneuronally the htau^FLAG−2N4RC291A^ transgene (dark grey bar), compared with the same transgene upon co-expression with Mical (black bar). Star indicates significant difference between the two genotypes. Control flies (light grey bars) are driver *elav*^*C155*^*-GAL4*/+ flies (CN) and flies that overexpress Mical. The number of experimental replicates (n) is indicated within the bars.
