# Supplementary material for: Mical modulates Tau toxicity via cysteine oxidation in vivo
Source: Acta Neuropathol Commun. 2022 Apr 4;10:44. doi: 10.1186/s40478-022-01348-1 (PMC8981811; doi:10.1186/s40478-022-01348-1)
Supplement: Supplementary file 1 — Additional file 1. Proteins interacting differentially with human Tau upon modulation of Mical levels. Selected proteins shown in alphabetical order, p-value and average log2 fold differences from three biological and three to four technical replicas have been calculated as described in Methods. The log2 fold change becomes positive when the affinity for Tau is increased and negative when it is decreased. The t-test was performed with a permutation-based FDR (False Discovery Rate of 0.05) calculation and the p-value determines the statistical significance. Table S1. Proteins implicated in microtubule cytoskeleton organization interacting differentially with human Tau upon Mical over-expression. Table S2. Proteins implicated in Actin cytoskeleton organization interacting differentially with human Tau upon Mical over-expression. Table S3. Proteins implicated in oxidation-reduction processes interacting differentially with human Tau upon Mical over-expression. Table S4. Proteins implicated in synaptic transmission interacting differentially with human Tau upon Mical over-expression. Table S5. Proteins implicated in cytoskeleton organization interacting differentially with human Tau upon Mical down-regulation. Selected proteins implicated in microtubule (upper group) and Actin (lower group) cytoskeleton organization are shown in alphabetical order. 14-3-3 epsilon does not interact differentially with Tau upon attenuation of Mical levels (p-value>0.05). The other proteins are differential Tau interactors when Mical is either up or down-regulated. Proteins in bold present opposite abundances in the two conditions. [file 40478_2022_1348_MOESM1_ESM.docx]

**Supplemental Table 1. Proteins implicated in microtubule cytoskeleton organization interacting differentially with human Tau upon Mical over-expression**.

| **Gene name** | **Identifier** | **Log2 Fold Change** | **p-value** |
| --- | --- | --- | --- |
| 14-3-3 eps epsilon | FBgn0020238 | 0,291649 | 1,73124E-07 |
| alpha-Spec | FBgn0250789 | 0,383806 | 0,000162127 |
| Ank2 | FBgn0261788 | 0,817559 | 1,379E-09 |
| awd | FBgn0000150 | -0,62705 | 0,013528569 |
| beta-Spec | FBgn0250788 | 0,753979 | 2,63763E-06 |
| Cam | FBgn0000253 | 2,475394 | 2,61161E-09 |
| Chc | FBgn0000319 | 1,087901 | 1,71969E-08 |
| Chro | FBgn0044324 | 1,030552 | 7,35316E-05 |
| Cp190 | FBgn0000283 | 0,737393 | 0,056165155 |
| ctp | FBgn0011760 | -0,72527 | 0,02539196 |
| dlg1 | FBgn0001624 | 0,242516 | 0,003418558 |
| Dmn | FBgn0021825 | -1,45122 | 0,000178762 |
| futsch | FBgn0259108 | 0,61213 | 0,001372934 |
| Grip84 | FBgn0026430 | 0,545579 | 0,005101283 |
| Grip91 | FBgn0001612 | 0,637491 | 0,000865773 |
| Jupiter | FBgn0051363 | -1,8945 | 1,5742E-08 |
| Khc | FBgn0001308 | 0,682157 | 0,005244579 |
| Khc-73 | FBgn0019968 | 0,799854 | 2,656E-07 |
| Pka-C1 | FBgn0000273 | -0,90729 | 7,78395E-05 |
| pont | FBgn0040078 | -1,11519 | 7,17172E-05 |
| Ran | FBgn0020255 | 0,613126 | 2,80615E-05 |
| Sac1 | FBgn0035195 | 0,964258 | 0,000128802 |
| sgg | FBgn0003371 | -0,33343 | 0,02912622 |
| shot | FBgn0013733 | -1,05731 | 0,026992274 |
| Synd | FBgn0053094 | -0,53598 | 0,003436243 |
| tacc | FBgn0026620 | -1,51795 | 0,025396898 |
| Tap42 | FBgn0051852 | -0,7781 | 0,003920212 |
| tau | FBgn0266579 | -8,97719 | 3,07325E-05 |
| Tcp-1zeta | FBgn0027329 | 0,494496 | 4,16554E-06 |
| tral | FBgn0041775 | -0,75019 | 0,000324472 |
| Vap-33A | FBgn0029687 | -0,76087 | 0,02826214 |
| wdb | FBgn0027492 | -0,82163 | 0,056231743 |

**Table S1.** Selected proteins shown in alphabetical order, p-value and average log2 fold differences from three biological and three to four technical replicas have been calculated as described in Methods. The log2 fold change becomes positive when the affinity for Tau is increased and negative when it is decreased. The t-test was performed with a permutation-based FDR (False Discovery Rate of 0.05) calculation and the p-value determines the statistical significance.

**Supplemental Table 2. Proteins implicated in Actin cytoskeleton organization interacting differentially with human Tau upon Mical over-expression**.

| **Gene name** | **Identifier** | **Log2 Fold Change** | **p-value** |
| --- | --- | --- | --- |
| ALiX | FBgn0086346 | -0,86923 | 0,012672577 |
| Arf51F | FBgn0013750 | -1,04757 | 0,006876016 |
| Arp1 | FBgn0011745 | -1,59923 | 0,00071432 |
| Arpc1 | FBgn0001961 | -0,97873 | 0,017108285 |
| Bsg | FBgn0261822 | -0,97817 | 0,037507693 |
| Cdc42 | FBgn0010341 | -1,38712 | 0,013752029 |
| CG32264 | FBgn0052264 | -0,88596 | 0,013008786 |
| coro | FBgn0265935 | -1,13422 | 1,07097E-07 |
| cpa | FBgn0034577 | -0,72342 | 2,34354E-05 |
| cpb | FBgn0011570 | -0,66489 | 0,000658649 |
| Galphao | FBgn0001122 | 0,410874 | 0,000351613 |
| Gel | FBgn0010225 | -0,69261 | 5,08284E-05 |
| jar | FBgn0011225 | -0,80472 | 0,052652715 |
| l(2)efl | FBgn0011296 | -0,59051 | 9,38844E-05 |
| Lam | FBgn0002525 | -1,8424 | 0,001099841 |
| LamC | FBgn0010397 | -1,31724 | 0,000664035 |
| Lasp | FBgn0063485 | -1,06626 | 0,05304292 |
| Mical | FBgn0053208 | 1,353622 | 0,021813351 |
| pnut | FBgn0013726 | -1,10917 | 9,72029E-06 |
| Prm | FBgn0003149 | -1,65529 | 0,00562594 |
| Rab11 | FBgn0015790 | 1,005172 | 0,001105517 |
| Rack1 | FBgn0020618 | 0,385685 | 9,01783E-05 |
| shi | FBgn0003392 | 0,64782 | 4,66684E-05 |
| sn | FBgn0003447 | -0,72069 | 0,009288416 |
| sqh | FBgn0003514 | -1,02577 | 0,000308142 |
| Tm1 | FBgn0003721 | -3,76393 | 6,56156E-07 |
| Tm2 | FBgn0004117 | -2,30048 | 0,000230566 |
| tmod | FBgn0082582 | -1,25303 | 0,002301536 |
| tsr | FBgn0011726 | 0,28183 | 0,004451179 |
| up | FBgn0004169 | -1,67994 | 0,037908914 |
| Vps4 | FBgn0027605 | 0,519317 | 0,054097903 |
| wupA | FBgn0000053 | -2,75468 | 1,18033E-07 |
| Zasp66 | FBgn0000053 | -2,75468 | 1,18033E-07 |

**Table S2.** Selected proteins shown in alphabetical order, p-value and average log2 fold differences from three biological and three to four technical replicas have been calculated as described in Methods. The log2 fold change becomes positive when the affinity for Tau is increased and negative when it is decreased. The t-test was performed with a permutation-based FDR (False Discovery Rate of 0.05) calculation and the p-value determines the statistical significance.

**Supplemental Table 3. Proteins implicated in oxidation-reduction processes interacting differentially with human Tau upon Mical over-expression**.

| **Gene name** | **Identifier** | **Log2 Fold** | **p-value** |
| --- | --- | --- | --- |
| 2HGDH | FBgn0032729 | 0,776971162 | 0,0088613 |
| ACC | FBgn0033246 | 0,470110055 | 0,0370609 |
| Acon | FBgn0010100 | 0,575777227 | 0,0001209 |
| aralar1 | FBgn0028646 | 1,007707422 | 0,0003876 |
| Atg3 | FBgn0036813 | 1,343980115 | 0,0013648 |
| BcDNA.GH10614 | FBgn0027552 | -0,574574904 | 0,0008598 |
| blw | FBgn0011211 | -0,268568444 | 0,0445157 |
| Ca-P60A | FBgn0263006 | 0,704367435 | 3,23E-06 |
| CG14407 | FBgn0030584 | -1,038415254 | 0,006242 |
| CG14997 | FBgn0035515 | 0,368190553 | 0,008302 |
| CG1544 | FBgn0039827 | -0,562683626 | 0,0346762 |
| CG17896 | FBgn0023537 | 0,280949968 | 0,012123 |
| CG2767 | FBgn0037537 | -0,597468521 | 0,0381969 |
| CG31075 | FBgn0051075 | 0,362871902 | 0,0013837 |
| CG32068 | FBgn0052068 | 1,239096651 | 0,0134552 |
| CG3603 | FBgn0029648 | 0,676329622 | 0,0180111 |
| CG3902 | FBgn0036824 | 0,571693266 | 0,0001158 |
| CG3999 | FBgn0037801 | -1,114616529 | 0,0047376 |
| CG5028 | FBgn0039358 | 0,803580005 | 0,0001067 |
| CG5653 | FBgn0035943 | -0,6180586 | 0,0154549 |
| CG6287 | FBgn0032350 | 0,571562931 | 0,0163856 |
| CG6523 | FBgn0032509 | 0,756670172 | 0,0340139 |
| CG7461 | FBgn0034432 | -0,594534402 | 0,0119251 |
| CG7675 | FBgn0038610 | -0,374415966 | 0,0150149 |
| CG7834 | FBgn0039697 | -0,588938068 | 6,11E-06 |
| CG8757 | FBgn0036380 | -1,700546727 | 0,0016274 |
| CG9331 | FBgn0032889 | -0,984160317 | 0,0012067 |
| CG9485 | FBgn0034618 | 0,745991312 | 0,0030077 |
| CG9547 | FBgn0031824 | -0,95426255 | 0,0427978 |
| CoVa | FBgn0019624 | -1,576668576 | 0,0107118 |
| COX5B | FBgn0031830 | 0,398215226 | 5,21E-05 |
| COX6B | FBgn0031066 | -1,387242943 | 0,0113736 |
| CPT2 | FBgn0035383 | -0,885415145 | 0,0018532 |
| CtBP | FBgn0020496 | -2,272713016 | 0,0009253 |
| Cyp4g1 | FBgn0010019 | 0,749246655 | 0,0515507 |
| Cyp9f2 | FBgn0038037 | 0,736514275 | 0,0110807 |
| cype | FBgn0015031 | -1,138752003 | 6,66E-11 |
| Cyt-c-p | FBgn0000409 | -3,251300985 | 4,95E-11 |
| Dbct | FBgn0030612 | -0,453551784 | 0,0005124 |
| Egm | FBgn0086712 | -0,609299689 | 0,0077044 |
| Gapdh1 | FBgn0001091 | 0,840681827 | 0,0406957 |
| Gapdh2 | FBgn0001092 | 0,896969998 | 5,90E-12 |
| Gdh | FBgn0001098 | 0,782307904 | 0,000106 |
| GlyP | FBgn0004507 | 0,265732351 | 0,0180016 |
| GlyS | FBgn0266064 | 0,367454105 | 0,012245 |
| Gpo-1 | FBgn0022160 | 0,441709846 | 0,0007099 |
| GstS1 | FBgn0010226 | 1,289278281 | 2,53E-07 |
| hgo | FBgn0040211 | -0,931637196 | 0,0165924 |
| Hn | FBgn0001208 | -0,633036662 | 5,23E-05 |
| Idh | FBgn0001248 | 0,592431791 | 0,0045233 |
| Idh3b | FBgn0038922 | 0,946328269 | 2,77E-06 |
| ImpL3 | FBgn0001258 | -0,278965806 | 0,0189767 |
| kdn | FBgn0261955 | 0,679983043 | 0,0001518 |
| l(1)G0156 | FBgn0027291 | 0,618325455 | 0,0016 |
| l(1)G0334 | FBgn0028325 | 0,413776783 | 0,0012109 |
| Mdh2 | FBgn0262559 | 0,970825388 | 0,0023113 |
| Men | FBgn0002719 | 0,468891375 | 0,0525054 |
| Men-b | FBgn0029155 | 0,623792764 | 3,68E-07 |
| Mical | FBgn0053208 | 1,353622437 | 0,0218134 |
| ND-13B | FBgn0047038 | -2,452569095 | 1,16E-07 |
| ND-15 | FBgn0031228 | -1,838947181 | 7,95E-07 |
| ND-18 | FBgn0031021 | -1,583153561 | 7,55E-08 |
| ND-19 | FBgn0035046 | -2,668104114 | 4,51E-08 |
| ND-20 | FBgn0030718 | 0,481046773 | 1,72E-05 |
| ND-24 | FBgn0030853 | 1,064357334 | 5,54E-11 |
| ND-49 | FBgn0039909 | 0,48200168 | 0,0043649 |
| ND-75 | FBgn0017566 | 0,656960073 | 6,52E-08 |
| ND-B14 | FBgn0033570 | -1,51568538 | 7,81E-09 |
| ND-B14.5A | FBgn0025839 | -2,553044117 | 7,25E-07 |
| ND-B17 | FBgn0001989 | -0,504938299 | 0,0120692 |
| ND-B17.2 | FBgn0031436 | -2,411148013 | 5,68E-05 |
| ND-B22 | FBgn0032511 | -1,581041182 | 0,0009981 |
| ND-MLRQ | FBgn0052230 | -3,994247745 | 0,0004666 |
| ox | FBgn0011227 | -1,953183068 | 0,0135427 |
| P5CDh1 | FBgn0037138 | 0,529260308 | 0,0203318 |
| P5cr | FBgn0015781 | -1,099851127 | 0,0003744 |
| P5cr-2 | FBgn0038516 | -0,591960888 | 3,42E-05 |
| ple | FBgn0005626 | 0,669445558 | 3,99E-05 |
| PPO1 | Bgn0283437 | 0,84199075 | 0,0002176 |
| PPO2 | FBgn0033367 | -0,662113883 | 0,0225924 |
| Prx2540-2 | FBgn0033518 | -0,552560806 | 1,18E-05 |
| Prx5 | FBgn0038570 | 0,30843334 | 0,0032842 |
| pug | FBgn0020385 | 0,40043723 | 0,0006878 |
| rdhB | FBgn0038946 | -0,697657633 | 0,0590086 |
| SdhB | FBgn0014028 | -0,547129776 | 0,000155 |
| sgll | FBgn0051472 | -0,285902004 | 0,0369216 |
| slgA | FBgn0003423 | 0,648446594 | 0,0003186 |
| Sod | FBgn0003462 | 1,376293394 | 1,48E-05 |
| Sod2 | FBgn0010213 | -0,733630807 | 0,0002008 |
| Sodh-1 | FBgn0024289 | -1,100853371 | 2,55E-05 |
| su(r) | FBgn0086450 | 0,361913604 | 0,0112791 |
| Sucb | FBgn0029118 | 0,361456572 | 0,0008419 |
| Trxr-1 | FBgn0020653 | -0,506166054 | 0,0226535 |
| Txl | FBgn0035631 | -0,373963269 | 0,0005145 |
| UGP | FBgn0035978 | -0,599816313 | 5,26E-05 |
| UQCR-14 | FBgn0030733 | -0,975671961 | 2,61E-09 |
| UQCR-C1 | FBgn0038271 | 0,437150165 | 5,83E-05 |
| UQCR-C2 | FBgn0250814 | 0,900809567 | 1,25E-09 |
| UQCR-Q | FBgn0036728 | -1,274815608 | 7,49E-08 |
| yip2 | FBgn0040064 | -0,321256772 | 0,0168168 |
| Zw | FBgn0004057 | -0,745623521 | 0,0270646 |

**Table S3.** Selected proteins shown in alphabetical order, p-value and average log2 fold differences from three biological and three to four technical replicas have been calculated as described in Methods. The log2 fold change becomes positive when the affinity for Tau is increased and negative when it is decreased. The t-test was performed with a permutation-based FDR (False Discovery Rate of 0.05) calculation and the p-value determines the statistical significance.

**Supplemental Table 4. Proteins implicated in synaptic transmission interacting differentially with human Tau upon Mical over-expression**.

| **Gene name** | **Identifier** | **Log2 Fold** | **p-value** |
| --- | --- | --- | --- |
| Ace | FBgn0000024 | -0,657040163 | 8,40303E-06 |
| Acsl | FBgn0263120 | 0,363587717 | 0,031002081 |
| AP-2sigma | FBgn0043012 | -1,463416629 | 0,026745633 |
| Arf102F | FBgn0013749 | -1,350787712 | 0,003156931 |
| Arf79F | FBgn0010348 | -0,419405985 | 0,00718286 |
| Atpalpha | FBgn0002921 | 0,945388235 | 2,06325E-06 |
| CaMKII | FBgn0264607 | 0,500151914 | 0,00412425 |
| CASK | FBgn0013759 | -0,969988698 | 0,005716074 |
| Clc | FBgn0024814 | -1,264128348 | 2,32656E-08 |
| comt | FBgn0000346 | 0,465031595 | 0,009503543 |
| EndoA;endoA | FBgn0038659 | 0,223759256 | 0,009258741 |
| Eps-15 | FBgn0035060 | 0,276121448 | 0,012267722 |
| Galphas | FBgn0001123 | 0,313110024 | 0,005128278 |
| gammaSnap2 | FBgn0266721 | -1,176775094 | 0,000156347 |
| lap | FBgn0086372 | 0,28582309 | 0,008812216 |
| nSyb | FBgn0013342 | -1,116292626 | 0,004353264 |
| pAbp | FBgn0265297 | 0,571472457 | 1,29662E-05 |
| Sap47 | FBgn0013334 | 1,190275308 | 1,6074E-06 |
| sesB | FBgn0003360 | 0,989685155 | 9,33865E-07 |
| Shab | FBgn0262593 | 1,396898616 | 3,46921E-06 |
| Shal | FBgn0005564 | 1,588237512 | 2,72816E-05 |
| Syn | FBgn0004575 | -2,549254273 | 0,003640681 |
| Synd | FBgn0053094 | -0,535983326 | 0,003436243 |
| Syt1 | FBgn0004242 | 0,769693529 | 4,14707E-06 |
| VAChT | FBgn0270928 | 0,568927611 | 0,003133488 |
| Vap-33A | FBgn0029687 | -0,760870211 | 0,02826214 |
| veli | FBgn0039269 | -1,189916225 | 2,87092E-11 |

**Table S4.** Selected proteins shown in alphabetical order, p-value and average log2 fold differences from three biological and three to four technical replicas have been calculated as described in Methods. The log2 fold change becomes positive when the affinity for Tau is increased and negative when it is decreased. The t-test was performed with a permutation-based FDR (False Discovery Rate of 0.05) calculation and the p-value determines the statistical significance.

**Supplemental Table 5. Proteins implicated in cytoskeleton organization interacting differentially with human Tau upon Mical down-regulation**.

| **Gene name** | **Identifier** | **Log2 Fold** | **p-value** |
| --- | --- | --- | --- |
| 14-3-3 epsilon | FBgn0020238 | -0,0379452 | 0,783303379 |
| alpha-Spec | FBgn0250789 | 0,282784 | 0,014697044 |
| **awd** | **FBgn0000150** | **0,785356** | **0,011763052** |
| **Chc** | **FBgn0000319** | **-0,548545** | **0,000845649** |
| **Cp190** | **FBgn0000283** | **-0,963162** | **0,015084121** |
| Khc | FBgn0001308 | 0,470451 | 0,029244891 |
| **pont** | **FBgn0040078** | **0,581807** | **0,01338166** |
| sgg | FBgn0003371 | -0,712173 | 0,000097947 |
| **shot** | **FBgn0013733** | **2,18905** | **0,000045807** |
| **Tap42** | **FBgn0051852** | **0,516713** | **0,001168234** |
| **tau** | **FBgn0266579** | **1,41476** | **0,010725561** |
| Tcp-1zeta | FBgn0027329 | 0,872341 | 0,002908439 |
|  |  |  |  |
| ALiX | FBgn0086346 | -2,44313 | 0,000030049 |
| **Arpc1** | **FBgn0001961** | **0,420409309** | **0,007257336** |
| coro | FBgn0265935 | -0,952666 | 0,00192349 |
| **cpa** | **FBgn0034577** | **0,481231** | **0,017053754** |
| **cpb** | **FBgn0011570** | **0,332262039** | **0,02266344** |
| **Gel** | **FBgn0010225** | **1,44502** | **0,000198802** |
| Galphao | FBgn0001122 | 0,579577 | 1,85E-06 |
| jar | FBgn0011225 | -0,936861 | 0,034497689 |
| l(2)efl | FBgn0011296 | -0,353812 | 0,038602906 |
| **Lasp** | **FBgn0063485** | **0,873271306** | **0,015091349** |
| **Prm** | **FBgn0003149** | **1,05422** | **0,00000138** |
| **Rack1** | **FBgn0020618** | **-0,492531** | **0,001223771** |
| **shi** | **FBgn0003392** | **-0,483171251** | **0,000200637** |
| sn | FBgn0003447 | -1,4346 | 0,000088196 |
| **Tm1** | **FBgn0003721** | **1,19328** | **0,000034774** |
| **Tm2** | **FBgn0004117** | **1,20237** | **0,000559551** |
| **up** | **FBgn0004169** | **0,911158** | **0,018344252** |
| **Vps4** | **FBgn0027605** | **-0,87023** | **0,000775104** |
| **wupA** | **FBgn0004028** | **0,73259** | **0,011294058** |

**Table S5.** Selected proteins implicated in microtubule (upper group) and Actin (lower group) cytoskeleton organization are shown in alphabetical order. 14-3-3 epsilon does not interact differentially with Tau upon attenuation of Mical levels (p-value>0.05). The other proteins are differential Tau interactors when Mical is either up or down-regulated. The proteins in bold present opposite abundances in the two conditions.
