# Supplementary figures and images for: Mical modulates Tau toxicity via cysteine oxidation in vivo
Source: Acta Neuropathol Commun. 2022 Apr 4;10:44. doi: 10.1186/s40478-022-01348-1 (PMC8981811; doi:10.1186/s40478-022-01348-1)

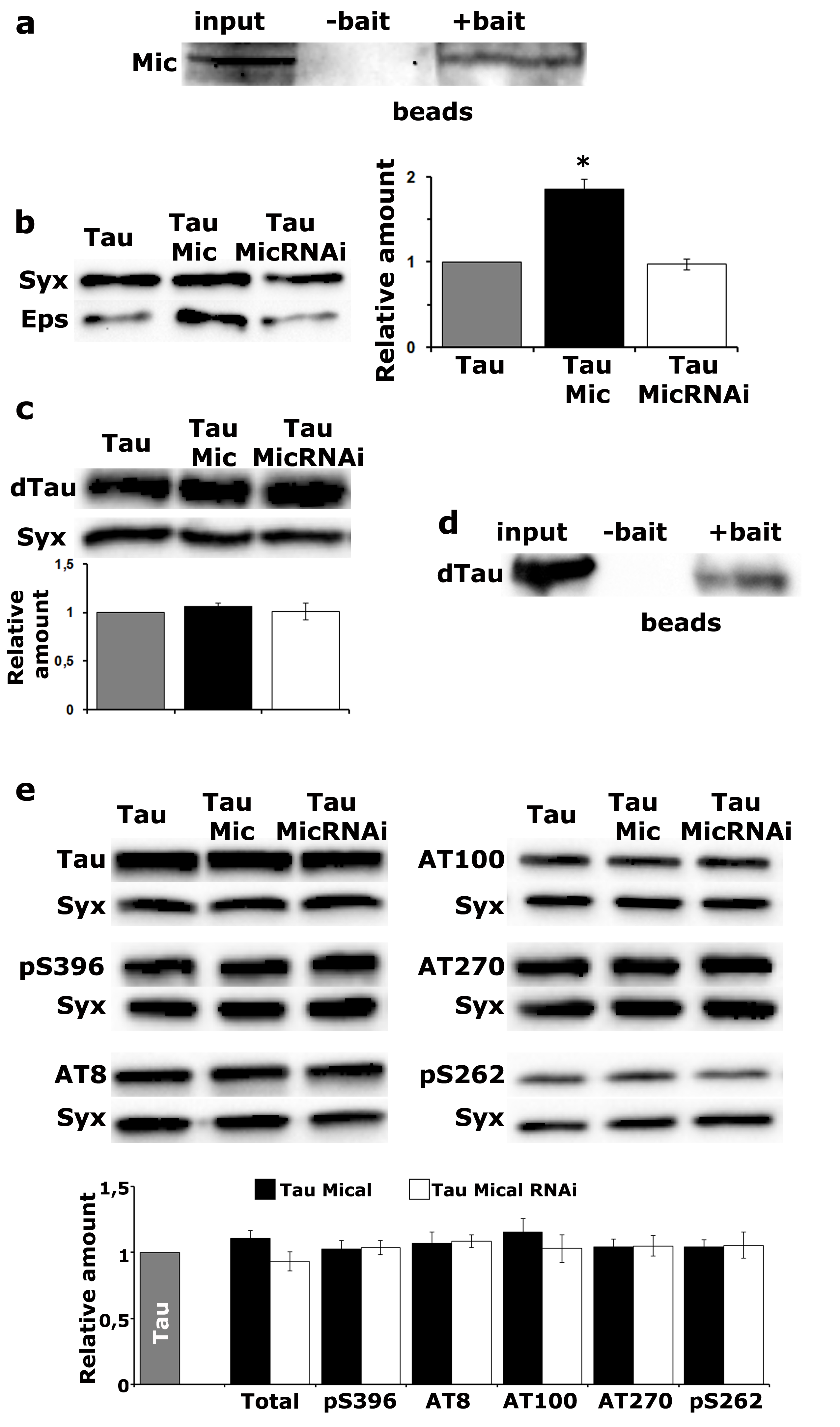

Supplement: Supplementary file 2 — Additional file 2: Fig. S1 a Verification of the Tau-Mical interaction via immunoblot analysis. Immunoprecipitation of htauFLAG−2N4R using anti-FLAG coated beads and subsequent western blot analysis using an anti-Mical antibody (+ bait). Anti-FLAG coated beads have equally been mixed with a lysate from flies overexpressing Mical in the absence of htauFLAG−2N4R to ensure non-specific binding of Mical to the beads (-bait). b Representative Western blots from head lysates of flies expressing Tau panneuronally compared with similar lysates co-expressing UAS-Mical or a UAS-Mical RNAi transgene probed for 14–3-3 epsilon and Syntaxin. The bars represent the mean ± SEM relative levels of 14–3-3 epsilon upon modulation of Mical levels. c Representative Western blots from head lysates of flies expressing Tau panneuronally compared with similar lysates co-expressing UAS-Mical or a UAS-Mical RNAi transgene probed for dTau and Syntaxin. The bars represent the mean ± SEM relative levels of dTau upon modulation of Mical levels. d Immunoprecipitation of htauFLAG−2N4R using anti-FLAG coated beads and subsequent western blot analysis using an anti-dTau antibody (+ bait). Anti-FLAG coated beads have equally been mixed with a lysate of elavC155-GAL4/+;Ras2-GAL4/+ flies to ensure non-specific binding of dTau to the beads (-bait). e Representative Western blots from head lysates of flies expressing Tau panneuronally compared with similar lysates co-expressing UAS-Mical or a UAS-Mical RNAi transgene probed with the indicated antibodies. Quantifications of four independent biological replicates are shown below in which levels of the phosphorylated protein were normalized using the Syntaxin (Syx) loading control. The normalized level of Tau expressed alone for each quantification was fixed to 1. The bars represent the mean ± SEM relative levels of Tau phosphorylated at the given site upon modulation of Mical levels over that of Tau expressed alone. [file 40478_2022_1348_MOESM2_ESM.tif]

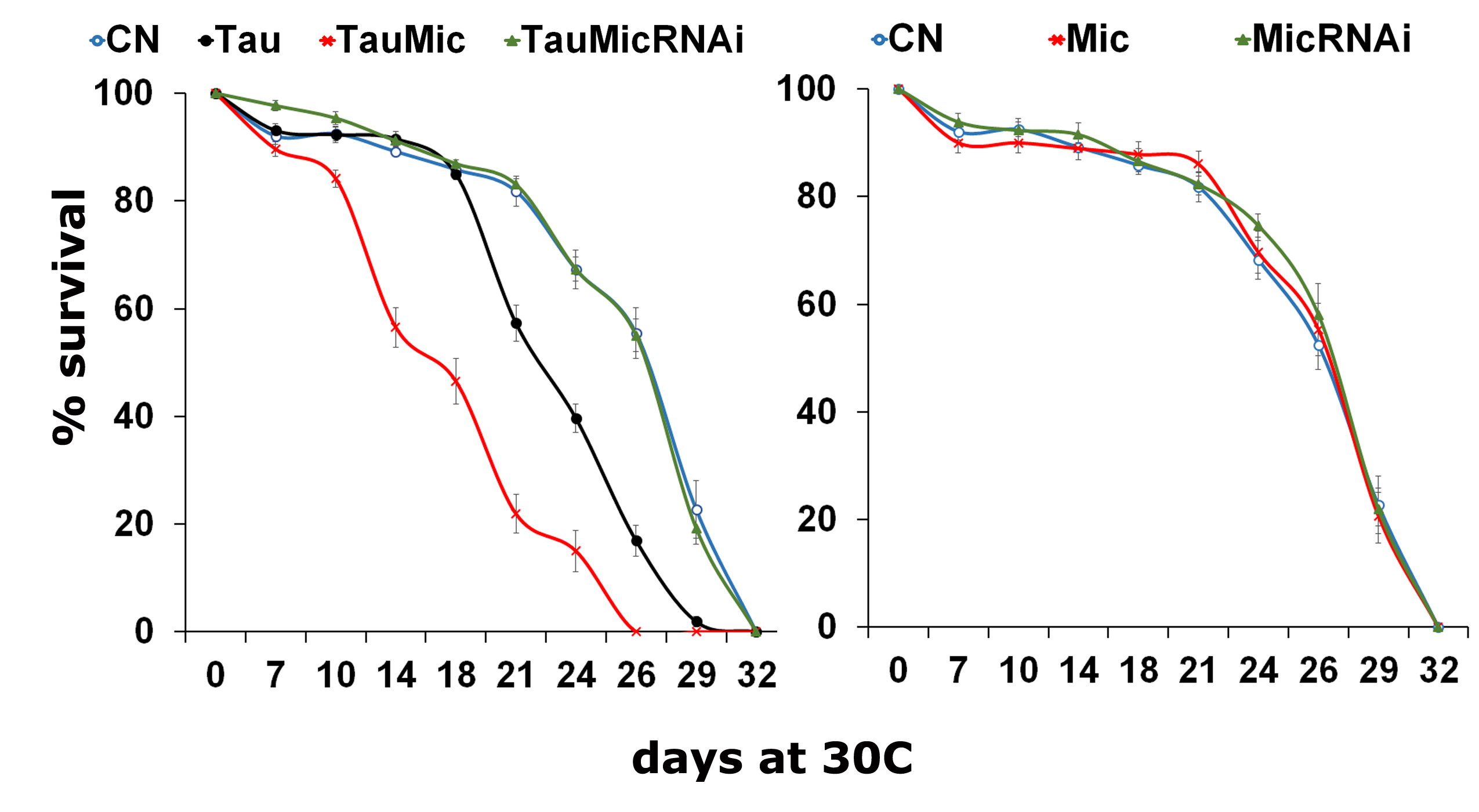

Supplement: Supplementary file 3 — Additional file 3: Fig. S2 Survival curves for male animals expressing panneuronally the indicated transgenes at 30 °C, in comparison with elavC155-GAL4/+;tub-Gal80ts/+ controls (CN). The data represent the mean ± SEM from two independent experiments with a total of 300 flies assessed per genotype. Statistical analysis indicated significant differences in longevity after accumulation of hTau0N4R alone and upon co-overexpression with Mical. Tau day 21 prob ChiSq = 0.00006 through day 29 prob ChiSq < 0.0002, TauMical day 10 prob ChiSq = 0.0009 through day 29 prob ChiSq < 0.00002 and TauMicRNAi prob ChiSq > 0.2. [file 40478_2022_1348_MOESM3_ESM.tif]

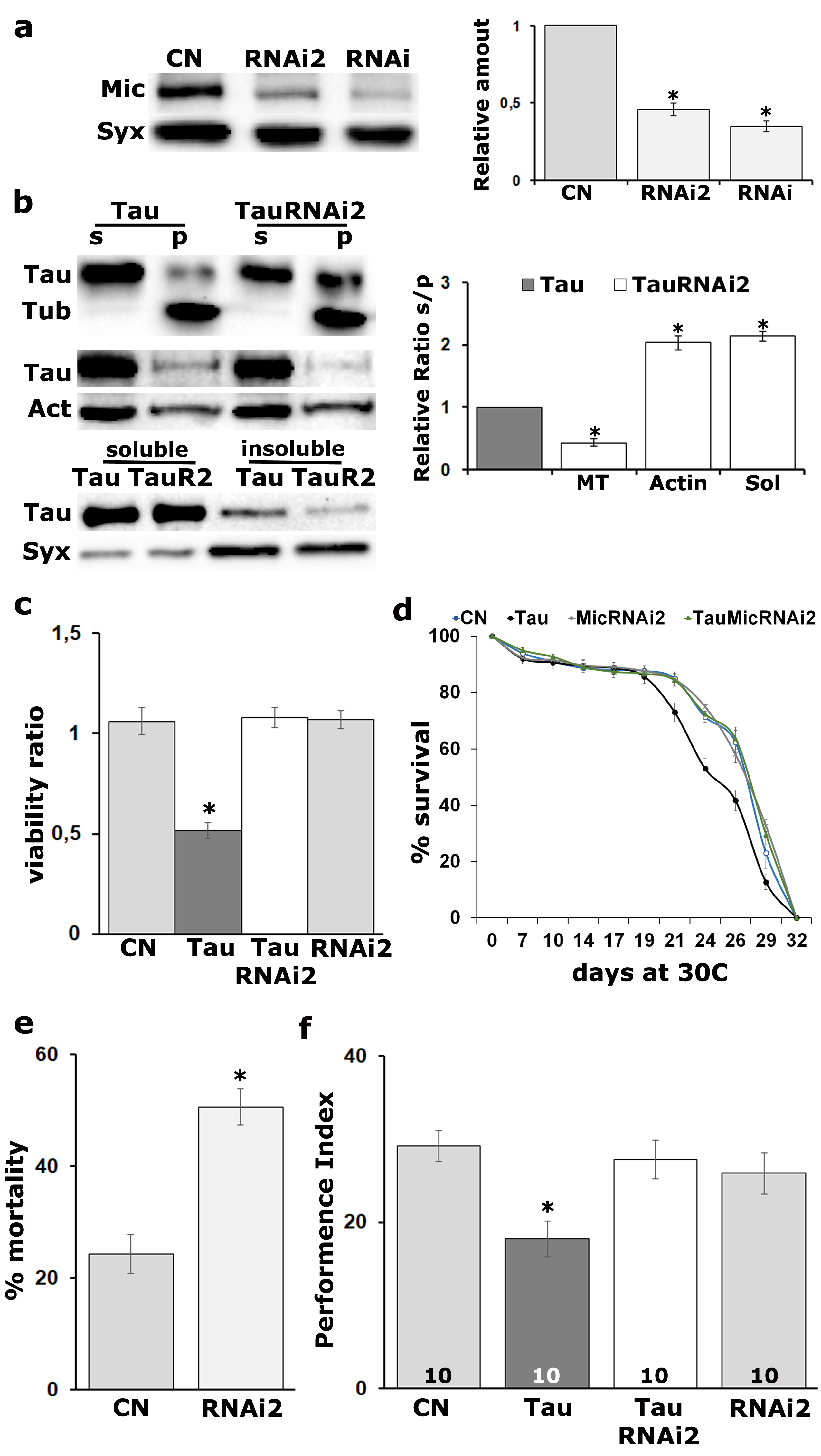

Supplement: Supplementary file 4 — Additional file 4: Fig. S3 Mical down-regulation with a second independent RNAi line. a Representative Western blot of head lysates from flies expressing UAS-Mical RNAi lines and probed with anti-Mical antibody. RNAi is line 18668R-2 and RNAi2 is line 25,372. The genotype of control animals was elavC155-GAL4/+. Stars indicate significant differences from control. b Endogenous microtubules (MT), phalloidin-bound F-Actin and aqueous soluble and insoluble fractions were isolated from flies expressing panneuronally the indicated transgenes. Pellet (p) and supernatant (s) fractions have been probed for Tau, Tubulin, Actin and Syntaxin respectively. Stars indicate significantly altered levels of precipitated Tau upon down-regulation of Mical levels (MT p = 0.0007, Actin p = 0.0006, sol p = 9.76e-0.5, n = 3). c Virgin female flies bearing the hTau0N4R transgene were crossed with elavC155-GAL4;UAS-MicRNAi2/CyO males. w1118 females were crossed with elavC155-GAL4 males (control progeny CN) and with elavC155-GAL4;UAS-MicRNAi2/CyO males to assess the viability of UAS-Mical RNAi2 transgene. Attenuation of Mical levels did not precipitate significant lethality on its own (MicRNAi2 vs control p = 0.9992, n = 6) but increased viability of Tau expressing animals to control levels (TauMicRNAi2 vs control p = 0.9876, n = 6). d Survival curves for animals expressing the indicated transgenes in comparison with elavC155-GAL4/+; tub-Gal80ts/+ controls. The life span of animals coexpressing hTau0N4R and Mical RNAi2 was statistically indistinguishable from that of controls (TauMicRNAi2 prob ChiSq > 0.3). e Response of flies expressing UAS-Mical RNAi2 upon treatment with paraquat for 28 h. Star indicates significant difference from control elavC155-GAL4/+ flies (p = 4.6905e−06). f Memory performance of animals expressing in the adult CNS for 12 days hTau0N4R alone and upon attenuation of Mical levels. Controls (light grey bars) were the elavC155-GAL4/+;tub-Gal80ts/+ and animals expressing t [file 40478_2022_1348_MOESM4_ESM.tif]

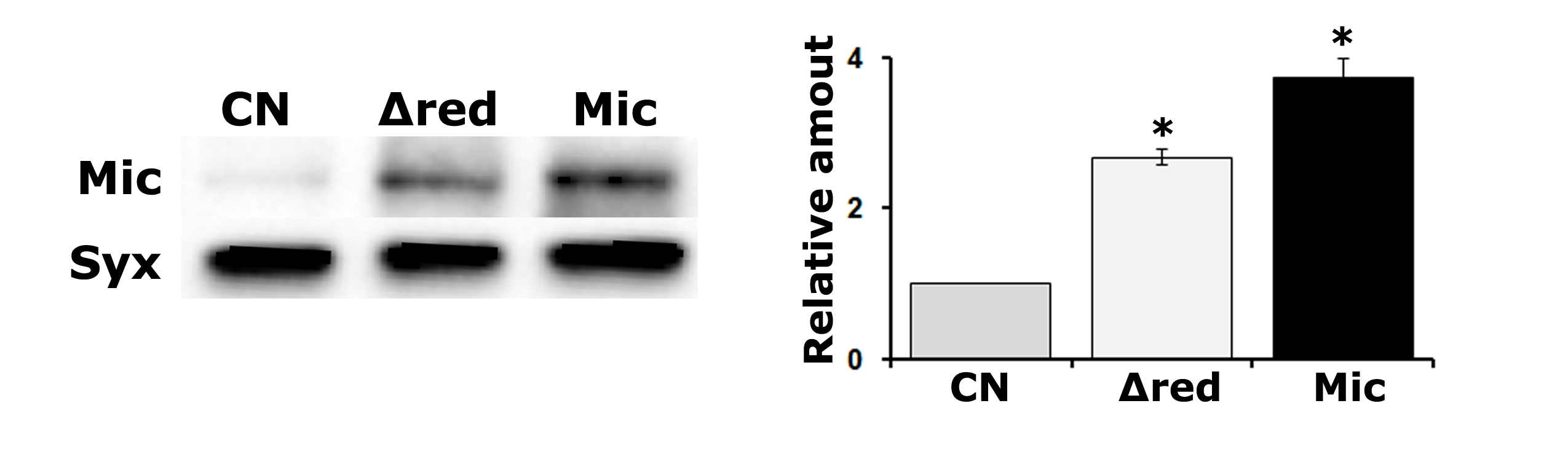

Supplement: Supplementary file 5 — Additional file 5: Fig. S4 UAS-Mical and UAS-MicalΔredox transgenes are expressed at comparable levels. Representative Western blot of head lysates from flies expressing the two UAS-Mical transgenic lines using elavC155-GAL4 and probed with anti-Mical antibody. The genotype of control animals was elavC155-GAL4/+. Stars indicate significant differences from control (CN). [file 40478_2022_1348_MOESM5_ESM.tif]

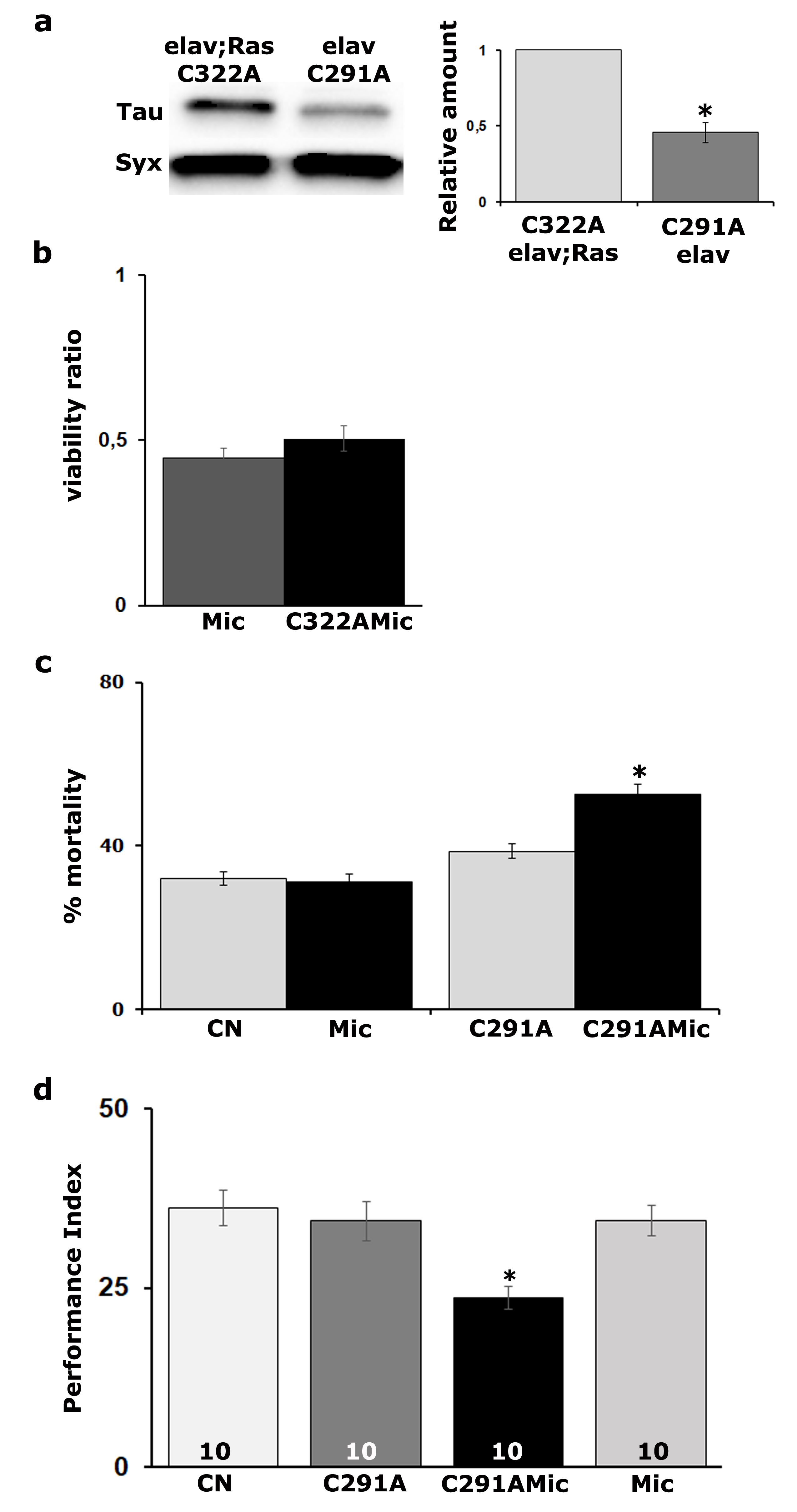

Supplement: Supplementary file 6 — Additional file 6: Fig. S5 Targeted proteomics to quantify cysteine oxidation. a Extracted chromatograms for the parent ions and isotopes (upper panel) and of its 6 most abundant fragments (daughter ions, lower panel) at the retention time 27.9 min of the NEM and carbamidomethyl labeled 322CGSLGNIHHKPGGGQVEVK peptide from representative samples of Tau and Tau co-overexpressed with Mical. b Spectra of the scan used for the library creation of the NEM (+ 125 Da) and carbamidomethyl (+ 57 Da) modified 322CGSLGNIHHKPGGGQVEVK peptide. [file 40478_2022_1348_MOESM6_ESM.tif]

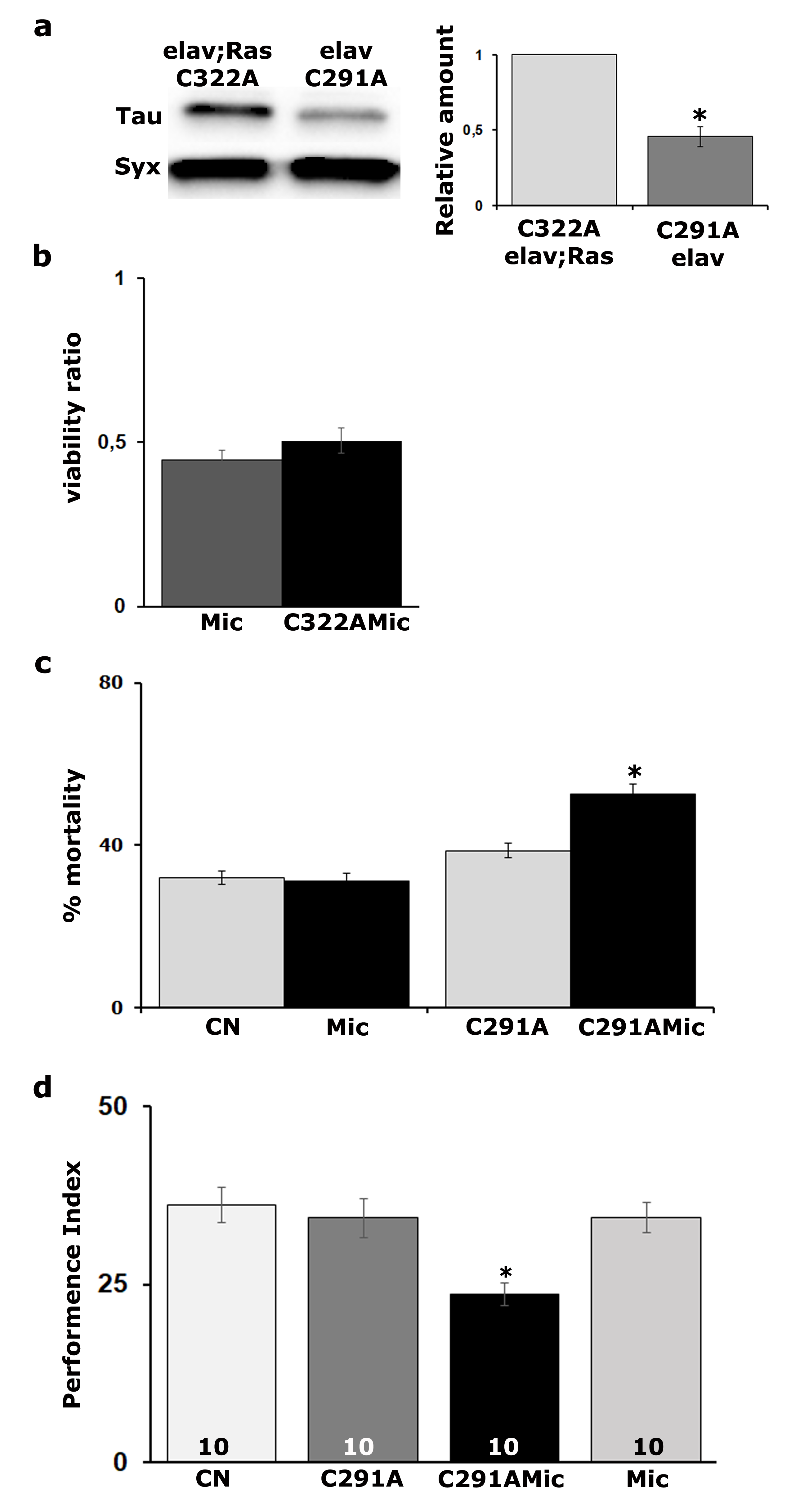

Supplement: Supplementary file 7 — Additional file 7: Fig. S6 a Representative Western blot of head lysates from flies expressing UAS-htauFLAG−2N4RC322A using elavC155-GAL4;Ras2-GAL4 and UAS-htauFLAG−2N4RC291A using elavC155-GAL4. Star indicates significant differences between the two groups. b Virgin elavC155-GAL4;Ras2-GAL4 females were crossed with UAS-Mic/CyO and UAS-Mic/CyO;UAS-C322A males. Bars represent the mean number of non-CyO bearing progeny over CyO flies ± SEM of the indicated genotypes. c Response of flies expressing UAS-htauFLAG−2N4RC291A upon treatment with paraquat for 28 h. Star indicates significant difference from the transgene without Mical overexpression. Control flies are elavC155-GAL4/+ (grey bar) and Mical are flies that overexpress Mical under the panneuronal driver (black bar). d Memory performance of animals expressing panneuronally the htauFLAG−2N4RC291A transgene (dark grey bar), compared with the same transgene upon co-expression with Mical (black bar). Star indicates significant difference between the two genotypes. Control flies (light grey bars) are driver elavC155-GAL4/+ flies (CN) and flies that overexpress Mical. The number of experimental replicates (n) is indicated within the bars. [file 40478_2022_1348_MOESM7_ESM.tif]
