# Supplementary figures and images for: Correction to: Mical modulates Tau toxicity via cysteine oxidation in vivo
Source: Acta Neuropathol Commun. 2022 Apr 25;10:63. doi: 10.1186/s40478-022-01369-w (PMC9040322; doi:10.1186/s40478-022-01369-w)

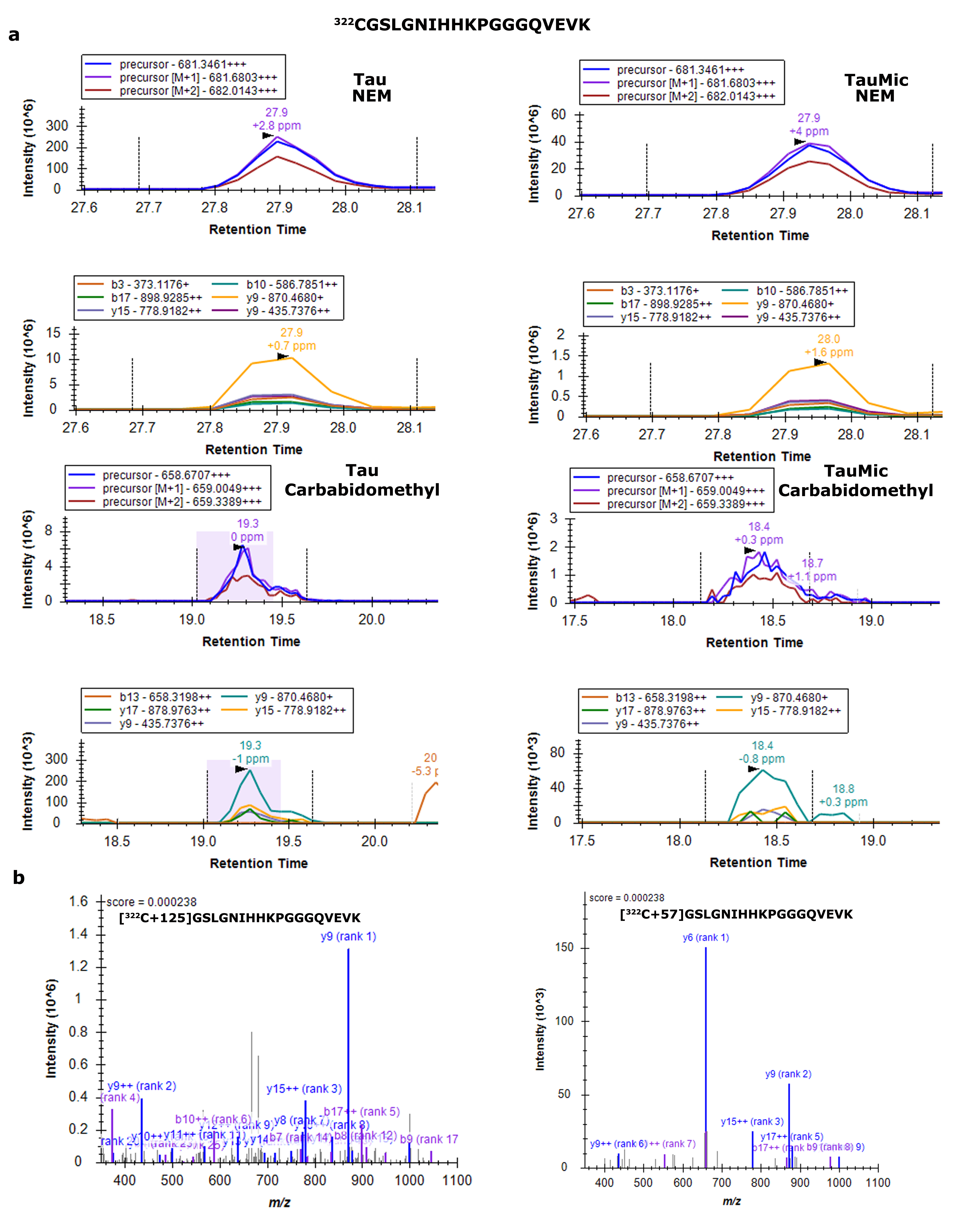

Supplement: Supplementary file 1 — Additional file 6: Fig. S5 Targeted proteomics to quantify cysteine oxidation. a Extracted chromatograms for the parent ions and isotopes (upper panel) and of its 6 most abundant fragments (daughter ions, lower panel) at the retention time 27.9 min of the NEM and carbamidomethyl labeled 322CGSLGNIHHKPGGGQVEVK peptide from representative samples of Tau and Tau co-overexpressed with Mical. b Spectra of the scan used for the library creation of the NEM (+ 125 Da) and carbamidomethyl (+ 57 Da) modified 322CGSLGNIHHKPGGGQVEVK peptide. [file 40478_2022_1369_MOESM6_ESM.tif]

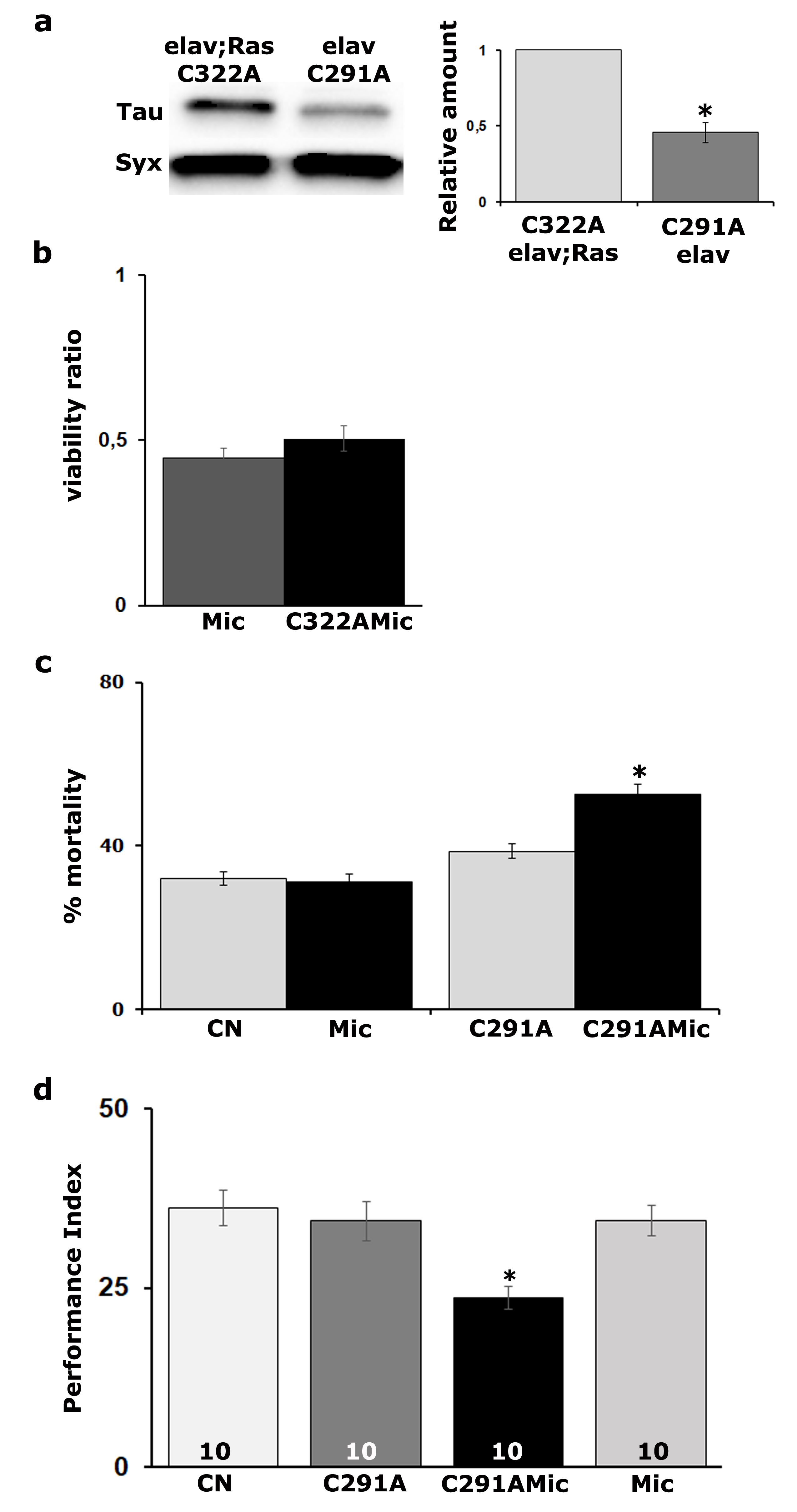

Supplement: Supplementary file 2 — Additional file 7: Fig. S6 a Representative Western blot of head lysates from flies expressing UAS-htauFLAG−2N4RC322A using elavC155-GAL4;Ras2-GAL4 and UAS-htauFLAG−2N4RC291A using elavC155-GAL4. Star indicates significant differences between the two groups. b Virgin elavC155-GAL4;Ras2-GAL4 females were crossed with UAS-Mic/CyO and UAS-Mic/CyO;UAS-C322A males. Bars represent the mean number of non-CyO bearing progeny over CyO flies ± SEM of the indicated genotypes. c Response of flies expressing UAS-htauFLAG−2N4RC291A upon treatment with paraquat for 28 h. Star indicates significant difference from the transgene without Mical overexpression. Control flies are elavC155-GAL4/+ (grey bar) and Mical are flies that overexpress Mical under the panneuronal driver (black bar). d Memory performance of animals expressing panneuronally the htauFLAG−2N4RC291A transgene (dark grey bar), compared with the same transgene upon co-expression with Mical (black bar). Star indicates significant difference between the two genotypes. Control flies (light grey bars) are driver elavC155-GAL4/+ flies (CN) and flies that overexpress Mical. The number of experimental replicates (n) is indicated within the bars. [file 40478_2022_1369_MOESM7_ESM.tif]
